# Polymorphisms in Genes of Relevance for Oestrogen and Oxytocin Pathways and Risk of Barrett’s Oesophagus and Oesophageal Adenocarcinoma: A Pooled Analysis from the BEACON Consortium

**DOI:** 10.1371/journal.pone.0138738

**Published:** 2015-09-25

**Authors:** Katarina Lagergren, Weronica E. Ek, David Levine, Wong-Ho Chow, Leslie Bernstein, Alan G. Casson, Harvey A. Risch, Nicholas J. Shaheen, Nigel C. Bird, Brian J. Reid, Douglas A. Corley, Laura J. Hardie, Anna H. Wu, Rebecca C. Fitzgerald, Paul Pharoah, Carlos Caldas, Yvonne Romero, Thomas L. Vaughan, Stuart MacGregor, David Whiteman, Lars Westberg, Olof Nyren, Jesper Lagergren

**Affiliations:** 1 Upper Gastrointestinal Surgery, Department of Molecular medicine and Surgery, Karolinska Institutet, Stockholm, Sweden; 2 Statistical Genetics, QIMR Berghofer Medical Research Institute, Herston, Queensland, Australia; 3 Department of Immunology, Genetics and Pathology, Science for Life Laboratory, Uppsala, Sweden; 4 Department of Biostatistics, University of Washington, Seattle, Washington, United States of America; 5 Department of Epidemiology, MD Anderson Cancer Center, Houston, Texas, United States of America; 6 Department of Population Sciences, Beckman Research Institute and City of Hope Comprehensive Cancer Center, Duarte, California, United States of America; 7 Department of Surgery, University of Saskatchewan, Saskatoon, SK, Canada; 8 Yale School of Public Health, New Haven, Connecticut, United States of America; 9 Division of Gastroenterology and Hepatology, UNC School of Medicine, University of North Carolina, Chapel Hill, North Carolina, United States of America; 10 Department of Oncology, The Medical School, University of Sheffield, Sheffield, United Kingdom; 11 Division of Human Biology, Fred Hutchinson Cancer Research Center, Seattle, Washington, United States of America; 12 Kaiser Permanente Northern California, Division of Research, Oakland, California, United States of America; 13 Division of Epidemiology, University of Leeds, Leeds, United Kingdom; 14 Department of Preventive Medicine, University of Southern California/Norris Comprehensive Cancer Center, Los Angeles, California, United States of America; 15 Medical Research Council (MRC) Cancer Unit, Hutchison-MRC Research Centre, University of Cambridge, Cambridge, United Kingdom; 16 Department of Public Health, University of Cambridge, Cambridge, United Kingdom; 17 Cancer Research UK Cambridge Institute, University of Cambridge, Cambridge, United Kingdom; 18 Division of Gastroenterology and Hepatology, Mayo Clinic, Rochester, Minnesota, United States of America; 19 Division of Public Health Sciences, Fred Hutchinson Cancer Research Center, Seattle, Washington, United States of America; 20 Cancer Control Group, Queensland Institute of Medical Research, Herston, Queensland, Australia; 21 Department of Pharmacology, Institute of Neuroscience and Physiology, Sahlgrenska Academy, University of Gothenburg, Gothenburg, Sweden; 22 Department of Medical Epidemiology and Biostatistics, Karolinska Institutet, Stockholm, Sweden; 23 Division of Cancer Studies, King’s College London, London, United Kingdom; Sapporo Medical University, JAPAN

## Abstract

**Background:**

The strong male predominance in oesophageal adenocarcinoma (OAC) and Barrett’s oesophagus (BO) continues to puzzle. Hormonal influence, e.g. oestrogen or oxytocin, might contribute.

**Methods:**

This genetic-epidemiological study pooled 14 studies from three continents, Australia, Europe, and North America. Polymorphisms in 3 key genes coding for the oestrogen pathway (receptor alpha (*ESR1*), receptor beta (*ESR2*), and aromatase (*CYP19A1*)), and 3 key genes of the oxytocin pathway (the oxytocin receptor (*OXTR*), oxytocin protein (*OXT*), and cyclic ADP ribose hydrolase glycoprotein (*CD38*)), were analysed using a gene-based approach, versatile gene-based test association study (VEGAS).

**Results:**

Among 1508 OAC patients, 2383 BO patients, and 2170 controls, genetic variants within *ESR1* were associated with BO in males (p = 0.0058) and an increased risk of OAC and BO combined in males (p = 0.0023). Genetic variants within *OXTR* were associated with an increased risk of BO in both sexes combined (p = 0.0035) and in males (p = 0.0012). We followed up these suggestive findings in a further smaller data set, but found no replication. There were no significant associations between the other 4 genes studied and risk of OAC, BO, separately on in combination, in males and females combined or in males only.

**Conclusion:**

Genetic variants in the oestrogen receptor alpha and the oxytocin receptor may be associated with an increased risk of BO or OAC, but replication in other large samples are needed.

## Introduction

Oesophageal adenocarcinoma (OAC) and its premalignant condition Barrett’s oesophagus (BO) have become increasingly common in the West during the last few decades.[1, 2] The up to 9:1 male-to-female ratio in OAC remains virtually unexplained.[1, 3]

### Oestrogen hypothesis

It has been hypothesised that the female sex hormone oestrogen may counteract the development of OAC, a hypothesis supported by a 20 year delay in the onset of this cancer in women compared to men,[4] and a particularly high male-to-female ratio during women’s reproductive years, compared to older ages.[5] A possible mechanism of oestrogen on OAC cells remains to be determined, but the presence of oestrogen receptors has repeatedly been shown in OAC,[6, 7] and a recent experimental study found that OAC and BO cells respond to treatment with selective oestrogen receptor ligands by decreased cell growth and apoptosis.[8] The hypothesis of oestrogen protection has, however, not been unequivocally supported in human studies on pharmacologically exposed individuals or on phenotypes reflecting presumed natural variation in oestrogen levels.[7] The low incidence of OAC in women and the uncertainty about the validity of assumptions concerning oestrogen exposure have been of major concern in previous studies.[3] In the absence of methods for assessment of the integrated steroid exposure given the diurnal and age-dependent within-person variation, assessment of genetic variants may be an alternative measure to assess oestrogen exposure.[9, 10] To the best of our knowledge, no previous study has addressed variants in genes known to regulate oestrogen levels in relation to risk of OAC or BO.

### Oxytocin hypothesis

As an increased duration of breastfeeding among women is associated with a substantially decreased risk of OAC,[11, 12] oxytocin is another conceivable mediator of the gender difference. Oxytocin levels are much higher in women than in men, and the hormone is richly released during breastfeeding.[13] Moreover, oxytocin receptors have been identified in the human gastrointestinal tract.[14] In addition, living without a partner is linked with an increased risk of OAC,[15] and oxytocin release is stimulated by physical contacts and interactions between people.[13] Higher oxytocin levels have also been shown to correlate with faster wound healing and less inflammation.[16] A plausible biological mechanism for any protective effect against OAC or BO is that the smooth muscle-contracting oxytocin[13] might raise the lower oesophageal sphincter pressure and counteract gastroesophageal reflux, the strongest known risk factor for OAC and BO.[17–19] We therefore hypothesised that high oxytocin activity might decrease the risk of OAC and BO. Oxytocin has a short half-life in serum, making serum level testing too unstable for research purposes, but polymorphisms in genes coding for oxytocin and its receptor might provide a marker of low oxytocin activity according to studies of behaviour and health in humans.[20–22]

To test the oestrogen and oxytocin hypotheses, we studied associations between single nucleotide polymorphisms (SNPs) in key genes coding for the oestrogen and oxytocin pathways in relation to the risks of OAC and BO.

## Materials and Methods

### Study design

Each study participant provided written informed consent to take part in the research, and the study was approved by the Fred Hutchinson Cancer Research Center Institutional Review Board in Seattle, WA. USA (number 7030, date 8/8/2014). We used harmonised data from the Barrett’s and Esophageal Adenocarcinoma Genetic Susceptibility Study (BEAGESS), a recent genome-wide association study (GWAS) conducted by the Barrett’s and Esophageal Adenocarcinoma Consortium (BEACON).[23] Included in the present analysis were all individuals contributed by investigators in the BEACON consortium to the BEAGESS. These individuals were of white-European ancestry, representing 14 cohort and case-control studies from three continents, Australia, Europe (England, Ireland and Sweden), and North America (Canada and United States) (S1 Text and S1 Table). Most of these studies were population-based, and have recently been included in pooled genetic studies.[23, 24] The data were used to study the association between SNPs in three key genes in the oestrogen pathway and three key genes in the oxytocin pathway. The selected genes in the oestrogen pathway were: 1) *ESR1*, coding for the oestrogen receptor alpha, 2) *ESR2*, coding for the oestrogen receptor beta, and 3) *CYP19A1*, coding for aromatase, an enzyme that catalyses the conversion of androgen to oestrogen. The selected genes coding for the oxytocin pathway included: 1) *OXTR*, coding for the oxytocin receptor, 2) *OXT*, coding for the oxytocin peptide, and 3) *CD38*, coding for the oxytocin secretion regulator cyclic ADP ribose hydrolase.[20] The SNPs included in the study are presented in S2 Table.

### Genotyping

Genotyping of DNA from buffy coat or whole blood was performed using the Illumina HumanOmni1-Quad platform. Annotations were based on version H of the Illumina product files and corresponded to the Genome Reference Consortium GRCh37 release. Samples with call rate <95% that either were an admixture of more than one DNA (n = 18), had low DNA input and a weak signal (n = 10), or were a noisy or poor quality sample (n = 4) were removed from further analysis. The remaining 6448 samples, including HapMap controls (n = 68) and duplicate samples (n = 67), underwent QA/QC steps as follows. We evaluated batch and plate effects using intensity data and allelic frequency and checked for case-control associations with different experimental factors. No important batch or plate effects or case-control associations with experimental factors were found. We used heterozygosity, sex chromosome intensity data, identity by descent (IBD) analysis and visualisation of B allele frequency (BAF) and log R ratio (LRR) plots to identify samples that had one or more of misannotated sex, unexpected relatedness, or were sample mixtures. Two sample mixtures were removed from further analysis. In the case of misannotated sex or unexpected relatedness, if the source of the discrepancy could be uncovered, the samples were kept; otherwise they were removed (n = 47) from further analysis. After further removing HapMap controls, duplicates and individuals with a missing call rate >2%, 6,061 BEAGESS samples remained for final analysis: 1,508 OAC cases, 2,383 BO cases, and 2,170 controls.

SNPs were excluded if they had a missing call rate >5%, Hardy Weinberg equilibrium p-value among controls ≤1e-4, a discordance among any of the duplicate pairs, a Mendelian error, or a minor allele frequency <1%. After QA/QC a total of 802,272 SNPs remained and were used for the initial GWAS analysis from which we selected 394 SNPs located within the selected genes for use in the versatile gene-based test association study (VEGAS) analysis described below.

### Association analysis

Case-control analyses were conducted with an additive logistic regression model where case status was regressed on each SNP genotype. OAC and BO were analysed separately, but since these conditions have a shared genetic background,[24] we also analysed a combined case category (phenotype OAC+BO) to increase power. The included covariates were sex, age and the first four principal components eigenvectors from a principal component analysis (PCA). The eigenvectors were included as covariates to account for population stratification due to ancestry. P-values for each case type (OAC, BO) or OAC and BO combined were calculated for each SNP and used as input for VEGAS. Data were also stratified by sex.

### Versatile Gene-Based Test Association Study (VEGAS)

VEGAS provides a gene-based approach that considers association between a trait and all SNPs within a specific gene or a subset of the most significant SNPs (for example the top 90% most significant SNPs),[25] rather than each SNP marker individually, as in a conventional GWAS. Even if the individual effect sizes at any given SNP are small, collectively, all SNPs within a gene could still account for a substantial proportion of variation in risk. Therefore, studies of combined risk alleles might identify candidate genes affecting disease. For some genes, an approach considering all SNPs within each gene might be the most powerful, but for some genes, considering only a subset of the most significant SNPs might be applicable. The true underlying genetic architecture is seldom known in advance and both approaches might be applicable to test. In this study, we first apply the full set of SNPs within each gene, and at a second step we also run VEGAS when including only the top 90% SNPs within each gene to remove non informative SNPs. Since the test including all SNPs and the one that included 90% top SNPs were highly correlated, we did not correct for this as being a new test. By combining the effects of all, or a subset of SNPs in a gene into a test statistic and correcting for linkage disequilibrium (LD), the gene-based test can assess combined effects between SNPs that would be missed in a GWAS. In this study, VEGAS was used to test whether there were any statistically significant effects for genes known to regulate oestrogen or oxytocin levels in OAC and BO patients. In brief, VEGAS explore associations on a per-gene basis using the p-values from all SNPs within a defined gene. An overlap of 10kb (upstream and downstream of the gene) for each gene was used. VEGAS corrects for LD as well as the number of SNPs within each gene. VEGAS takes account of LD between markers in a gene by using simulation based on the LD structure of a set of reference individuals, or, as in this study, using a custom set of individuals whose genotype information was available.[26] A Bonferroni corrected p-value of 0.006 was considered statistically significant since we run 9 tests for each hormone (0.05/9 = 0.0056).

## Results

### Study participants

Selected characteristics of the study participants, 1508 OAC case patients, 2383 BO case patients, and 2170 control participants, are presented in Table 1. The distributions of sex and age were similar between these case groups and the control group.

**Table 1:** Characteristics of oesophageal adenocarcinoma cases (OAC), Barrett’s oesophagus cases (BO), either of these (OAC+BO) and control subjects.

	BO	OAC	OAC+BO	Controls
	*Number (%)*	*Number (%)*	*Number (%)*	*Number (%)*
**Total**	2383 (100)	1508 (100)	3891 (100)	2170 (100)
***Men***	1808 (76)	1333 (88)	3141 (81)	1704 (79)
***Women***	575 (24)	175 (12)	750 (19)	466 (21)
**Age groups (both sexes)**				
***<50***	401 (17)	125 (8)	526 (14)	304 (14)
***50–59***	630 (26)	365 (24)	995 (25)	551 (26)
***60–69***	618 (26)	496 (33)	1114 (29)	745 (34)
***≥70***	734 (31)	522 (35)	1256 (32)	570 (26)

#### Polymorphisms in the oestrogen pathway

When including all SNPs within each gene in the gene-based test for the un-stratified data, none of the 3 genes tested showed significant association in both sexes combined (Table 2). However, sex stratified analysis revealed that genetic variants within the gene coding for the oestrogen receptor alpha (*ESR1*) indicated an increased risk of OAC and BO combined in males (p = 0.0081) (Table 3). When including only the top 90% SNPs within each gene using the VEGAS test, variants in *ESR1* were possibly associated with an increased risk of BO and OAC combined in males and females (p = 0.0063) (Table 4). In males, a corresponding potential association was found with BO (p = 0.0058), which was statistically significant with BO and OAC combined (p = 0.0023) (Table 5). The most significant SNP was rs2982684 (located in intron 4), while most other significant SNPs were located in introns 3 and 4 (S3 Table and S4 Table). Polymorphisms in the other two studied genes in the oestrogen pathway did not reach a statistically significant level of association with BO, OAC or OAC or BO, independent of sex stratification or inclusion of top 90% SNPs within each gene (Tables 2 and 3; Tables 4 and 5).

**Table 2:** Gene-based analysis of genes known to regulate oestrogen levels and oxytocin levels and risk of Barrett’s oesophagus (BO), oesophageal adenocarcinoma (OAC), and these conditions combined (OAC+BO). P-values in bold are statistically significant after correction for multiple testing.

					P-value		
Gene	Chr^1^	SNPs (N)	Start position (bp^2^)	Stop position (bp^2^)	BO	OAC	OAC+BO
***ESR1***	6	224	152011630	152424408	0.067	0.042	0.034
***ESR2***	14	36	64693750	64805267	0.54	0.73	0.59
***CYP19A1***	15	60	51500253	51630794	0.40	0.66	0.54
***OXT***	20	10	3052265	3053162	0.96	**0.50**	0.99
***OXTR***	3	35	8792093	8811299	0.019	**0.67**	0.13
***CD38***	4	29	15779920	15850705	0.38	**0.28**	0.34

^1^ Chromosome number

^2^ base pair.

**Table 3:** Sex-specific gene-based analysis for genes known to regulate oestrogen levels and oxytocin levels and risk of Barrett’s oesophagus (BO), oesophageal adenocarcinoma (OAC), and these conditions combined (OAC+BO). P-values in bold are statistically significant.

			p-values females			p-values males		
Gene	Chr^1^	SNPs (N)	BO^a^	OAC^b^	OAC+BO^c^	BO^d^	OAC^e^	OAC+BO^f^
***ESR1***	6	224	0.26	0.73	0.32	0.032	0.043	0.015
***ESR2***	14	36	0.69	0.37	0.90	0.33	0.59	0.37
***CYP19A1***	15	60	0.17	0.22	0.25	0.18	0.81	0.49
***OXT***	20	10	0.88	0.84	0.93	0.99	0.59	0.99
***OXTR***	3	35	0.83	0.90	0.78	0.0081	0.57	0.067
***CD38***	4	29	0.75	0.75	0.69	0.38	0.25	0.29

^1^ Chromosome number

^a^ 575 cases and 466 controls

^b^ 175 cases and 466 controls

^c^ 751 cases and 466 controls

^d^ 1808 cases and 1703 controls

^e^ 1333 cases and 1704 controls, and

^f^ 3141 cases and 1702 controls.

**Table 4:** Gene-based analysis of top 90% single nucleotide polymorphisms (SNPs) in genes known to regulate oestrogen levels and oxytocin levels and risk of Barrett’s oesophagus (BO), oesophageal adenocarcinoma (OAC), and these conditions combined (OAC+BO). P-values in bold are statistically significant after correction for multiple testing.

Gene	Chr^1^	SNPs (N)	Start position (bp^2^)	Stop position (bp^2^)	BO 90%	OAC 90%	OAC+BO 90%
***ESR1***	6	224	152011630	152424408	0.015	0.0075	0.0063
***ESR2***	14	36	64693750	64805267	0.40	0.58	0.43
***CYP19A1***	15	60	51500253	51630794	0.24	0.45	0.35
***OXT***	20	10	3052265	3053162	0.96	0.50	0.99
***OXTR***	3	35	8792093	8811299	**0.0035**	0.41	0.037
***CD38***	4	29	15779920	15850705	0.26	**0.17**	0.22

^1^ Chromosome number

^2^ base pair.

**Table 5:** Sex-specific gene-based analysis of top 90% single nucleotide polymorphisms (SNPs) in genes known to regulate oestrogen levels and oxytocin levels and risk of Barrett’s oesophagus (BO), oesophageal adenocarcinoma (OAC), and these conditions combined (OAC+BO). P-values in bold are statistically significant after correction for multiple testing.

			p-values females			p-values males		
Gene	Chr^1^	SNPs (N)	BO^a^	OAC^b^	OAC+BO^c^	BO^d^	OAC^e^	OAC+BO^f^
***ESR1***	6	224	0.082	0.37	0.11	**0.0058**	0.0087	**0.0023**
***ESR2***	14	36	0.56	0.23	0.82	0.21	0.43	0.23
***CYP19A1***	15	60	0.075	0.11	0.12	0.086	0.62	0.31
***OXT***	20	10	0.88	0.84	0.93	0.99	0.59	0.99
***OXTR***	3	35	0.59	0.69	0.52	**0.0012**	0.30	0.017
***CD38***	4	29	0.62	0.60	0.54	0.24	0.15	0.19

^1^ Chromosome number

^a^ 575 cases and 466 controls

^b^ 175 cases and 466 controls

^c^ 751 cases and 466 controls

^d^ 1808 cases and 1703 controls

^e^ 1333 cases and 1704 controls, and

^f^ 3141 cases and 1702 controls.

#### Polymorphisms in the oxytocin pathway

When including all SNPs within each gene in the gene-based test for the non-stratified data, none of the 3 genes tested in the oxytocin pathways showed significant association after correcting for multiple testing (Table 2). However, the sex stratified analyses showed that genetic variants within the oxytocin receptor gene (*OXTR*) were statistically borderline associated with BO in males (p = 0.0081). Moreover, when including only the top 90% SNPs within each gene, *OXTR* variants were significantly associated to BO in both sexes combined (p = 0.0035) and in males separately (p = 0.0012) (Tables 4 and 5). The most significant *OXTR* SNP was rs237902 (S5 Table). Polymorphisms in the other two studied genes in the oxytocin pathway did not reach a statistically significant level of association with BO, OAC or OAC or BO, independent of sex stratification or inclusion of top 90% SNPs within each gene (Tables 2 and 3; Tables 4 and 5).

## Discussion

This study demonstrated possible associations between SNPs in *ESR1* and risk of BO and OAC, and SNPs in *OXTR* and risk of BO. The other four genes tested, i.e. *ESR2* and *CYP19A1* in the oestrogen pathway and *OXT* and *CD38* in the oxytocin pathway, did not show any statistically significant association with OAC or BO.

Strengths of this study include the population-based design of the included studies, the extensive data on genetic variants through the assessment of SNPs of relevant genes, and a sample size that exceeds that in most previous studies concerned with BO and OAC. Yet, the low number of female cases of OAC and BO makes it difficult to assess potential associations in females only or to ascertain any potential differences in associations between men and women. Chance findings from multiple testing is a threat to many genetic studies, but our strictly defined hypotheses and the selection of analysis of only three key genes for each hypothesis counteract such errors. Moreover, all results were corrected for multiple testing in the statistical analyses. We used Bonferroni correction for the number of genes tested for each hormone, which is an established method in this respect, and such approach does not need the same low p-value as for a GWAS that needs to correct for 1M tests. Nevertheless, chance cannot be dismissed as a potential explanation for the positive associations identified. Limited statistical power might be the reason for the lack of statistically significant associations among females. A fraction of variance for each gene will remain unexplained, to which rare variants may contribute. To discover rare variants and test them for association with a phenotype, a follow up study could re-sequence a small initial sample size and then genotype the discovered variants in a larger sample set. Finally, the study was based on individuals of white-European ancestry, and the results might not be generalizable to other populations.

Gene-based tests for association are increasingly being seen as useful complements to GWAS.[25] A gene-based approach considers association between a trait and all markers, or a subset of markers, within a specific gene rather than each marker individually, as in a GWAS. VEGAS assigns SNPs to each of 17,787 autosomal genes according to positions on the UCSC Genome Browser hg19 assembly. To capture regulatory regions and SNPs in LD, we defined gene boundaries as 10 kb. Depending on the underlying genetic architecture, gene-based approaches can be more powerful than traditional individual SNP-based GWAS. For example, if a gene contains more than one causative variant, several SNPs within that gene might show marginal effects that are often indistinguishable from random noise in the GWAS results. However, under some genetic architecture, a more powerful gene based method may be to consider only the top most significant SNPs in a gene rather than the full set of SNPs. In this study, we applied both these methods and confirmed a significant effect for *ESR1* and *OXTR* when including only the top 90% SNPs for each gene.

There are, to the best of our knowledge, no previous genetic studies that have addressed the hypotheses tested in the present study. The biological effects of oestrogen are mediated by two distinct oestrogen receptors, alpha and beta, and these exist in both sexes.[27] These receptors often exert opposite effects on cellular processes that differentially influence the development and the progression of cancer.[28] The oestrogen receptor alpha, of particular relevance in this study, is associated with aberrant proliferation, inflammation and cancer development.[28] The finding of an association between SNPs in the gene coding for the oestrogen receptor alpha and an association to BO is therefore interesting. This finding suggests the possibility that a functioning oestrogen pathway might act against OAC development, and therefore possibly be involved in explaining the lower incidence of this tumour in females. There were, however, a too limited number of female cases to assess potential differences in associations between the sexes. Nevertheless, although oestrogen levels are lower in men than women, the results indicate that the oestrogen pathway might be involved in the aetiology of malignant progression also in males. Previous research has found a role for genetic variations in the *ESR1* gene in determining post-menopausal plasma oestrogen levels in women.[10]

The finding of an increased risk of BO with variants of the *OXTR* is in line with the study hypothesis. The oxytocin receptor binds to G proteins which enables oxytocin to activate multiple responses in the cell. The oxytocin system, i.e. oxytocin and the oxytocin receptor, can influence the growth modulation of various neoplastic cells by stimulating or inhibiting cell proliferation, depending on the characteristics and conditions of the cell. Oxytocin can inhibit proliferation of neoplastic cells of other epithelial origin, e.g. in the ovary,[29] endometrium,[30] prostate,[31] bone[32], breast,[33] and in neuroblastoma and glial tumours.[34] Oxytocin can increase the intracellular concentration of cAMP resulting in decreased proliferation, a mechanism that might be relevant for the association with BO in the present study, but further research is needed. It is also not proven that the investigated SNPs in the oxytocin pathway actually affect the serum levels or activity of this hormone, although previous research indicates that such gene variants can influence oxytocin activity in humans,[20–22] which lends some support for this genetic approach to assess levels of oxytocin as exposure.

Regarding single SNP-associations it seems likely that the association from the gene-based analysis of *ESR1* and risk of OAC or BO in men is mainly due to the most significant SNP, rs2982684, located in intron 4, together with other SNPs spanning introns 3 and 4. Intriguingly in this context is that several previous studies have reported associations between SNPs in intron 4 of *ESR1* and oestrogen-dependent traits,[35–39] including breast cancer risk.[36] The most significant *OXTR* SNP in the present study, rs237902 is located in exon 3 and has previously been associated with behavioural phenotypes,[40, 41] as well as susceptibility to preterm birth.[42] However, the functional relevance of this SNP remains to be clarified.

The results of the present study require confirmation in future studies based on very large sample sizes. In the absence of an at least equally large sample population, we tried to replicate our positive findings in an independent cohort from the United Kingdom, including 851 BO cases and 977 OAC cases (47% of the total case number of the present study), and 2785 control subjects from the 1958 British Birth Cohort who were genotyped on a custom version of the Illumina Human1.2M-Duo array.[43] This array comprised less SNPs in each of the candidate genes compared to the Illumina HumanOmni-1-Quad array used for the BEAGESS sample. We analysed the replication dataset in a similar fashion as the main study, but none of the associations between SNPs in *ESR1* and *OXTR* in relation to risk to BO or OAC replicated. The lack of replication might be due to that the positive findings from the main dataset were a result of chance errors. However, the lack of replication was not entirely unexpected due to the limited size of the replication sample. Thus, replication in a larger sample is needed.

In conclusion, this large-scale study of polymorphisms in genes coding for the oestrogen and oxytocin pathways provides some evidence that variants in genes coding the oestrogen receptor alpha and the oxygen receptor might be associated with the risk of BO, OAC, and BO or OAC. These findings indicate a possible hormonal influence in the sex difference in incidence of BO and OAC, but the results need to be cautiously interpreted and require confirmation in future large-scale studies.

## Supporting Information

S1 Text (DOCX)

S1 Table (DOCX)

S2 Table (DOCX)

S3 Table (DOCX)

S4 Table (DOCX)

S5 Table (DOCX)

S6 Table (DOCX)

## Abbreviations

CYP19A1: Aromatase
BO: Barrett’s oesophagus
CD38: Cyclic ADP ribose hydrolase glycoprotein
OAC: Oesophageal adenocarcinoma
ESR1: Oestrogen receptor alpha
ESR2: Oestrogen receptor beta
GWAS: Genome-wide association study
OXT: Oxytocin protein
OXTR: Oxytocin receptor
VEGAS: Versatile gene-based test association study
