# Supplementary material for: Polymorphisms in Genes of Relevance for Oestrogen and Oxytocin Pathways and Risk of Barrett’s Oesophagus and Oesophageal Adenocarcinoma: A Pooled Analysis from the BEACON Consortium
Source: PLoS One. 2015 Sep 25;10(9):e0138738. doi: 10.1371/journal.pone.0138738 (PMC4583498; doi:10.1371/journal.pone.0138738)
Supplement: S1 Text — (DOCX) [file pone.0138738.s001.docx]

**Supporting Information**

**S1 Text:**

**Subject populations**

**Oesophageal adenocarcinoma and Barrett’s oesophagus**

Specifically; two population based studies from the Queensland Institute of Medical Research[^1^](#_ENREF_1)^,^[^2^](#_ENREF_2); one population based study from the University of Southern California[^3^](#_ENREF_3); a Kaiser Permanente, Northern California community-based case-control study[^4^](#_ENREF_4); a Nova Scotia, Canada case-control study based on cases seen at a tertiary referral centre[^5^](#_ENREF_5)^,^[^6^](#_ENREF_6); The US Multicenter Study, a population-based case-control study involving three areas, New Jersey, Connecticut and western Washington state, in a cooperative agreement with NCI[^7-9^](#_ENREF_7); a simultaneous population-based study of OAC and BO from Ireland (Republic and Northern), using hospital, clinic and pathology records, with a shared control group representing the population from a province-wide database of practitioner records included [^10^](#_ENREF_10)^,^[^11^](#_ENREF_11); a population-based Swedish study of OAC cases and representative population controls selected from the population registry [^12^](#_ENREF_12); a tertiary clinic-based English case-control study of OAC and BO, with non-esophagitis clinic controls[^13^](#_ENREF_13); a Toronto, Canada hospital-based case-control study of OAC using controls selected from case friends or spouse of other cancer cases[^14^](#_ENREF_14); a prospective cohort of consecutive patients with long segment BO and OAC recruited by a multi-campus, multidisciplinary, tertiary care academic consortium since September 10, 2001, located in Rochester, MN, Scottsdale, AZ, and Jacksonville, FL[^15-17^](#_ENREF_15); a University of North Carolina study of BO and GERD controls[^18^](#_ENREF_18); a western Washington community-clinic based study of newly diagnosed BO cases and population controls[^19^](#_ENREF_19); and a western Washington cohort of persons with BO under active surveillance for the development of OAC from the Seattle Barrett’s Esophagus Research Program [^20^](#_ENREF_20)^,^[^21^](#_ENREF_21) .

OAC cases had clinically diagnosed and histologically confirmed OAC. BO patients had histologically confirmed intestinal metaplasia and endoscopically evident columnar epithelium in the tubular oesophagus (a more detailed definition of BO for each study can be found in Supplementary Table 1). Samples were genotyped on the Illumina Omni1-Quad array. Quality control procedures removed low-quality and incorrectly identified samples. Further, related individuals were identified and only one individual from each family was used in any particular analysis.

**References**

**1.** Whiteman D, Sadeghi S, Pandeya N, et al. Combined effects of obesity, acid reflux and smoking on the risk of adenocarcinomas of the oesophagus. *GUT.* 2008;57(2):173-180.

**2.** Smith K, O'Brien S, Smithers B, et al. Interactions among smoking, obesity, and symptoms of acid reflux in Barrett's esophagus. *Cancer Epidemiol Biomarkers Prev.* 2005;11(14):2481-2486.

**3.** Wu A, Wan P, Bernstein L. A multiethnic population-based study of smoking, alcohol and body size and risk of adenocarcinomas of the stomach and esophagus. *Cancer Causes Control.* 2001;12(8):721-732.

**4.** Corley D, Kubo A, Levin T, et al. Abdominal obesity and body mass index as risk factors for Barrett's esophagus. *Gastroenterol.* 2007;133(1):34-41.

**5.** Casson A, Zheng Z, Evans S, Veugelers P, Porter G, Guernsey D. Polymorphisms in DNA repair genes in the molecular pathogenesis of esophageal (Barrett) adenocarcinoma. *Cacriogenesis.* 2005;26(9):1536-1541.

**6.** Veugelers P, Porter G, Guernsey D, Casson A. Obesity and lifestyle risk factors for gastroesophageal reflux disease, Barrett esophagus and esophageal adenocarcinoma. *Dis Esophagus.* 2006;19(5):321-328.

**7.** Engel L, Chow W, Vaughan T, et al. Population attributable risks of esophageal and gastric cancers. *J Natl Cancer Inst.* 2003;95(18):1404-1413.

**8.** Chow W, Blot W, Vaughan T, et al. Body mass index and risk of adenocarcinomas of the esophagus and gastric cardia. *J Natl Cancer Inst.* 1998;90(2):150-155.

**9.** Gammon M, Schoenberg J, Ahsan H, et al. Tobacco, alcohol, and socioeconomic status and adenocarcinomas of the esophagus and gastric cardia. *J Natl Cancer Inst.* 1997;89(17):1277-1284.

**10.** Anderson L, Johnston B, Watson R, et al. Nonsteroidal anti-inflammatory drugs and the esophageal inflammation-metaplasia-adenocarcinoma sequence. *Cancer Res.* 2006;66(9):4975-4982.

**11.** Corley D, Kerlikowske K, Verma R, Buffler P. Protective association of aspirin/NSAIDs and esophageal cancer: a systematic review and meta-analysis. *Gastroenterol.* 2003;124(1):47-56.

**12.** Lagergren J, Bergström R, Lindgren A, Nyrén O. Symptomatic gastroesophageal reflux as a risk factor for esophageal adenocarcinoma. *N Engl J Med.* 1999;340(11):825-831.

**13.** Martino Ed, Hardie L, Wild C, et al. The NAD(P)H:quinone oxidoreductase I C609T polymorphism modifies the risk of Barrett esophagus and esophageal adenocarcinoma. *Genet Med.* 2007;9(6):341-347.

**14.** Zhai R, Liu G, Asomaning K, et al. Genetic polymorphisms of VEGF, interactions with cigarette smoking exposure, and esophageal adenocarcinoma risk. *Cacriogenesis.* 2008;29(12):2330-2334.

**15.** Miller RC, Atherton PJ, Kabat BF, et al. Marital status and quality of life in patients with esophageal cancer or Barrett esophagus: A Mayo Clinic Esophageal Adenocarcinoma and Barrett’s Esophagus Registry Study. *Dig Dis Sci* 2010;55(10):2860-2868.

**16.** Gatenby PA, Caygill CP, Watson A, Murray L, Romero Y. Barrett’s esophagus registries. *Ann N Y Acad Sci* 2011;1232(1):405-410.

**17.** Stauder MC, Romero Y, Kabat B, et al. Overall survival and self-reported fatigue in patients with esophageal cancer. *Support Care Cancer.* 2012.

**18.** Shaheen N, Green B, Medapalli R, et al. The perception of cancer risk in patients with prevalent Barrett's esophagus enrolled in an endoscopic surveillance program. *Gastroenterol.* 2005;129(2):429-436.

**19.** Edelstein Z, Farrow D, Bronner M, Rosen S, Vaughan T. Central adiposity and risk of Barrett's esophagus. *Gastroenterol.* 2007;133(2):403-411.

**20.** Galipeau P, Li X, Blount P, et al. NSAIDs modulate CDKN2A, TP53, and DNA content risk for progression to esophageal adenocarcinoma. *PLoS Med.* 2007;4(2):e67.

**21.** Lin D. Evaluating statistical significance in two-stage genomewide association studies. *Am J Hum Genet.* 2006;78:505-509.
