# Supplementary material for: Polymorphisms in Genes of Relevance for Oestrogen and Oxytocin Pathways and Risk of Barrett’s Oesophagus and Oesophageal Adenocarcinoma: A Pooled Analysis from the BEACON Consortium
Source: PLoS One. 2015 Sep 25;10(9):e0138738. doi: 10.1371/journal.pone.0138738 (PMC4583498; doi:10.1371/journal.pone.0138738)
Supplement: S1 Table — (DOCX) [file pone.0138738.s002.docx]

**Supporting Information**

**S2 Table.** Participants from each study site for the GWAS data used, including cases of oesophageal adenocarcinoma (OAC), cases of Barrett’s oesophagus (BO), and control subjects.

|  | | | n genotyped (n excluded) | | |  | |
| --- | --- | --- | --- | --- | --- | --- | --- |
| Location | | Study/ Reference | OAC cases | BO cases | Controls | Total Genotyped | Total Analysed |
| Australia | |  |  |  |  |  |  |
|  | Nationwide |  | 236 | 0 | 245 | 481 | 481 |
|  | Queensland, Australia |  | 0 | 326^1^ (1) | 323 (5) | 655 | 649 |
| Subtotal | |  | 236 | 326 (1) | 568 (5) | 1136 | 1130 |
| Europe | |  |  |  |  |  |  |
|  | Sheffield, UK |  | 102 | 167^2^ (7) | 0 | 276 | 269 |
|  | Sweden-wide |  | 64 | 0 | 116 (1) | 181 | 180 |
|  | Ireland, Republic of Ireland |  | 194 | 199^3^ | 218 (2) | 613 | 611 |
| Subtotal | |  | 360 | 366 (7) | 334 (3) | 1070 | 1060 |
| North America | |  |  |  |  |  |  |
|  | Kaiser Permanente, Northern California, US |  | 0 | 242^4^ (30) | 215 (30) | 517 | 457 |
|  | Washington & New Jersey, US | EGA study | 56 | 0 | 114 (2) | 172 | 170 |
|  | Rochester, Minnesota, US | Mayo registry | 503 (2) | 814^5^ (5) | 0 | 1324 | 1317 |
|  | Toronto, Ontario, Canada |  | 248 (23) | 0 | 259 (13) | 543 | 507 |
|  | Raleigh, North Carolina, US |  | 0 | 100^6^ (1) | 0 | 101 | 100 |
|  | Washington, US | Study Reflux Disease | 0 | 157^7^ (3) | 167 | 327 | 324 |
|  | Washington, US | Seattle Barrett’s Oesophagus Program | 0 | 296^8^ (6) | 0 | 302 | 296 |
|  | Nova Scotia, Canada |  | 54 | 115^9^ (6) | 92 (1) | 268 | 261 |
|  | Los Angeles, California, US |  | 60 (1) | 0 | 438 (6) | 505 | 498 |
| Subtotal | |  | 921 (26) | 1724 (51) | 1285 (52) | 4059 | 3930 |
| Total BEACON consortium | |  | 1517 (26) | 2416 (59) | 2187 (60) | 6265 | 6120 |

^1^BO was defined as the presence of specialised intestinal metaplasia (columnar epithelium with goblet cells) in a biopsy taken from the oesophagus by upper gastrointestinal endoscopy, regardless of the length of involvement. Patients with specialised intestinal metaplasia detected only in biopsies taken from the gastric cardia were not eligible for inclusion.

^2^BO was defined as any length of histologically confirmed specialised intestinal metaplasia containing goblet cells.

^3^BO patients were eligible for inclusion if ≥3 cm of typical Barrett's mucosa was seen at endoscopy, and the presence of specialised intestinal metaplasia was confirmed by histologic examination of biopsy specimens. Patients with dysplasia on histologic examination were not included.

^4^BO was defined if the endoscopist clearly described a visible length of columnar-type epithelium proximal to the gastroesophageal junction/gastric fold and if a biopsy showed specialised intestinal epithelium. Pathology slides underwent a separate manual review by a gastrointestinal pathologist. The following patients were excluded: patients with only gastric-type metaplasia of the oesophagus on all pathologic evaluations, patients with columnar metaplasia without features of intestinal metaplasia on all pathology readings, patients without a biopsy specimen of oesophageal origin, biopsy specimens of only a mildly irregular squamo-columnar junction.

^5^BO was defined as long segment (≥3 cm), histologically confirmed specialized intestinal metaplasia containing goblet cells.

^6^BO was defined as any detectable upward displacement of the squamo-columnar junction into the tubular oesophagus, with at least one biopsy specimen showing columnar epithelium with goblet cells. Patients with goblet cells on biopsy examination but no endoscopic appearance of BO were not eligible for inclusion.

^7^During the endoscopy procedure, the physicians recorded the presence or absence of visible columnar epithelium and, if present, its length. Based on these findings, cases were subsequently classified into 1, 2, or 3 of the following progressively exclusive groups: (1) BO cases (i.e., all cases), (2) BO cases with visible columnar epithelium (visible Barrett’s oesophagus) and (3) BO cases with visible column epithelium greater than 2 cm (long-segment BO).

^8^BO was diagnosis as metaplastic columnar epithelium with intestinal metaplasia in oesophageal biopsies, the absence of oesophageal malignancy at or prior to baseline endoscopy and having had at least one follow-up endoscopy.

^9^The diagnosis of BO was established by the histological finding of intestinal metaplasia, which was confirmed independently by two consultant gastrointestinal histopathologists.
