# Supplementary material for: Polymorphisms in Genes of Relevance for Oestrogen and Oxytocin Pathways and Risk of Barrett’s Oesophagus and Oesophageal Adenocarcinoma: A Pooled Analysis from the BEACON Consortium
Source: PLoS One. 2015 Sep 25;10(9):e0138738. doi: 10.1371/journal.pone.0138738 (PMC4583498; doi:10.1371/journal.pone.0138738)
Supplement: S2 Table — (DOCX) [file pone.0138738.s003.docx]

**Supporting Information**

**S3 Table.** Single nucleotide polymorphisms (SNPs) included for the 3 studied genes in the oestrogen pathway (*ESR1, ESR2, CYP19A1*) and 3 studied genes in the oxytocin pathway (OXT, OXTR, CD38) used in VEGAS.

| **Gene** | **Chromosome** | **SNP** | **Base pair position** |
| --- | --- | --- | --- |
| ESR1 | 6 | rs6557164 | 152002932 |
| ESR1 | 6 | rs851995 | 152005534 |
| ESR1 | 6 | rs851993 | 152006011 |
| ESR1 | 6 | rs9383939 | 152006178 |
| ESR1 | 6 | rs851991 | 152006581 |
| ESR1 | 6 | rs851987 | 152007889 |
| ESR1 | 6 | kgp10311820 | 152008442 |
| ESR1 | 6 | rs3020331 | 152008780 |
| ESR1 | 6 | kgp3926446 | 152008924 |
| ESR1 | 6 | kgp6713052 | 152009427 |
| ESR1 | 6 | rs2941740 | 152009638 |
| ESR1 | 6 | rs3020333 | 152010254 |
| ESR1 | 6 | kgp6718446 | 152010377 |
| ESR1 | 6 | kgp12313848 | 152010534 |
| ESR1 | 6 | kgp2093333 | 152010561 |
| ESR1 | 6 | kgp11248855 | 152012739 |
| ESR1 | 6 | rs3020334 | 152012956 |
| ESR1 | 6 | kgp737782 | 152012988 |
| ESR1 | 6 | kgp1921179 | 152013748 |
| ESR1 | 6 | kgp9736427 | 152013760 |
| ESR1 | 6 | kgp11394755 | 152016803 |
| ESR1 | 6 | rs1293936 | 152017691 |
| ESR1 | 6 | kgp2922863 | 152018139 |
| ESR1 | 6 | kgp3955218 | 152018498 |
| ESR1 | 6 | kgp705069 | 152020390 |
| ESR1 | 6 | kgp1417894 | 152021049 |
| ESR1 | 6 | kgp9300867 | 152023191 |
| ESR1 | 6 | kgp10100871 | 152024178 |
| ESR1 | 6 | rs851983 | 152024415 |
| ESR1 | 6 | rs851982 | 152024985 |
| ESR1 | 6 | kgp17127196 | 152027012 |
| ESR1 | 6 | kgp2475865 | 152027074 |
| ESR1 | 6 | kgp5788762 | 152027955 |
| ESR1 | 6 | kgp6899289 | 152028755 |
| ESR1 | 6 | rs851978 | 152029556 |
| ESR1 | 6 | kgp5232249 | 152031303 |
| ESR1 | 6 | kgp9690084 | 152031496 |
| ESR1 | 6 | kgp11951474 | 152039385 |
| ESR1 | 6 | kgp9635185 | 152039444 |
| ESR1 | 6 | kgp9033598 | 152039889 |
| ESR1 | 6 | kgp5012159 | 152039964 |
| ESR1 | 6 | kgp3691071 | 152040125 |
| ESR1 | 6 | kgp10208621 | 152040257 |
| ESR1 | 6 | rs12525163 | 152040291 |
| ESR1 | 6 | kgp9534209 | 152040615 |
| ESR1 | 6 | rs10484921 | 152042260 |
| ESR1 | 6 | kgp10486525 | 152042413 |
| ESR1 | 6 | kgp1144405 | 152042502 |
| ESR1 | 6 | kgp11513921 | 152043290 |
| ESR1 | 6 | rs1159327 | 152048022 |
| ESR1 | 6 | kgp2380438 | 152048217 |
| ESR1 | 6 | kgp6354401 | 152049501 |
| ESR1 | 6 | kgp2855478 | 152051854 |
| ESR1 | 6 | kgp6631336 | 152052043 |
| ESR1 | 6 | kgp7957493 | 152052601 |
| ESR1 | 6 | kgp11024871 | 152052652 |
| ESR1 | 6 | rs3020343 | 152054363 |
| ESR1 | 6 | rs12195741 | 152054374 |
| ESR1 | 6 | kgp12208636 | 152055606 |
| ESR1 | 6 | kgp6494875 | 152056081 |
| ESR1 | 6 | kgp1691518 | 152056146 |
| ESR1 | 6 | kgp1103762 | 152056368 |
| ESR1 | 6 | kgp5296648 | 152057408 |
| ESR1 | 6 | rs3020348 | 152057914 |
| ESR1 | 6 | kgp5641093 | 152058010 |
| ESR1 | 6 | kgp8324978 | 152058844 |
| ESR1 | 6 | rs2982552 | 152059563 |
| ESR1 | 6 | kgp10613698 | 152059787 |
| ESR1 | 6 | rs2982551 | 152061210 |
| ESR1 | 6 | kgp3397459 | 152062530 |
| ESR1 | 6 | kgp8113620 | 152063998 |
| ESR1 | 6 | kgp2765867 | 152064199 |
| ESR1 | 6 | kgp5408731 | 152064355 |
| ESR1 | 6 | kgp3560501 | 152064454 |
| ESR1 | 6 | kgp2861858 | 152064464 |
| ESR1 | 6 | kgp3189538 | 152064487 |
| ESR1 | 6 | rs3020306 | 152065886 |
| ESR1 | 6 | rs1856057 | 152067869 |
| ESR1 | 6 | rs1999805 | 152068364 |
| ESR1 | 6 | kgp8502758 | 152068591 |
| ESR1 | 6 | kgp4577083 | 152068685 |
| ESR1 | 6 | kgp8802865 | 152068874 |
| ESR1 | 6 | kgp8372881 | 152069791 |
| ESR1 | 6 | kgp9144294 | 152069999 |
| ESR1 | 6 | kgp11639523 | 152072718 |
| ESR1 | 6 | kgp2911751 | 152075487 |
| ESR1 | 6 | kgp3916874 | 152080039 |
| ESR1 | 6 | kgp4245848 | 152081009 |
| ESR1 | 6 | rs1336981 | 152082369 |
| ESR1 | 6 | kgp5179039 | 152082646 |
| ESR1 | 6 | kgp584372 | 152082973 |
| ESR1 | 6 | rs9479109 | 152084593 |
| ESR1 | 6 | kgp6294659 | 152084862 |
| ESR1 | 6 | kgp8301700 | 152084973 |
| ESR1 | 6 | rs1890010 | 152085275 |
| ESR1 | 6 | kgp7859664 | 152087111 |
| ESR1 | 6 | kgp7324582 | 152087318 |
| ESR1 | 6 | kgp8702359 | 152088155 |
| ESR1 | 6 | rs2485209 | 152089768 |
| ESR1 | 6 | kgp10166489 | 152090024 |
| ESR1 | 6 | rs6939257 | 152090045 |
| ESR1 | 6 | rs6939683 | 152090324 |
| ESR1 | 6 | kgp7561639 | 152090535 |
| ESR1 | 6 | rs2504063 | 152090707 |
| ESR1 | 6 | rs9371553 | 152091428 |
| ESR1 | 6 | kgp4921988 | 152092638 |
| ESR1 | 6 | rs4870053 | 152092749 |
| ESR1 | 6 | kgp1077733 | 152094838 |
| ESR1 | 6 | kgp11619301 | 152095043 |
| ESR1 | 6 | rs2504065 | 152095167 |
| ESR1 | 6 | rs2248586 | 152095332 |
| ESR1 | 6 | kgp9819860 | 152095694 |
| ESR1 | 6 | kgp7268606 | 152095829 |
| ESR1 | 6 | rs2504067 | 152095987 |
| ESR1 | 6 | rs17828471 | 152097405 |
| ESR1 | 6 | kgp9235060 | 152098571 |
| ESR1 | 6 | kgp6725383 | 152098687 |
| ESR1 | 6 | kgp1450269 | 152101823 |
| ESR1 | 6 | rs528529 | 152102939 |
| ESR1 | 6 | rs1285057 | 152103792 |
| ESR1 | 6 | rs543650 | 152110943 |
| ESR1 | 6 | rs17081685 | 152116655 |
| ESR1 | 6 | rs2881766 | 152119119 |
| ESR1 | 6 | rs11964281 | 152121442 |
| ESR1 | 6 | rs488133 | 152125444 |
| ESR1 | 6 | rs2071454 | 152126824 |
| ESR1 | 6 | rs2077647 | 152129077 |
| ESR1 | 6 | rs532010 | 152130918 |
| ESR1 | 6 | rs3853248 | 152139886 |
| ESR1 | 6 | rs9371557 | 152140209 |
| ESR1 | 6 | rs3844509 | 152140464 |
| ESR1 | 6 | rs7759411 | 152148870 |
| ESR1 | 6 | rs11969288 | 152149200 |
| ESR1 | 6 | rs11155813 | 152149435 |
| ESR1 | 6 | rs7761133 | 152151863 |
| ESR1 | 6 | rs6909023 | 152153697 |
| ESR1 | 6 | rs827423 | 152156197 |
| ESR1 | 6 | rs827421 | 152157122 |
| ESR1 | 6 | rs6902771 | 152157881 |
| ESR1 | 6 | rs9322331 | 152162317 |
| ESR1 | 6 | rs2234693 | 152163335 |
| ESR1 | 6 | rs827419 | 152177663 |
| ESR1 | 6 | rs1643821 | 152183551 |
| ESR1 | 6 | rs11155818 | 152184130 |
| ESR1 | 6 | rs1709183 | 152193996 |
| ESR1 | 6 | rs11155819 | 152199359 |
| ESR1 | 6 | rs9322335 | 152200129 |
| ESR1 | 6 | rs9322336 | 152200430 |
| ESR1 | 6 | rs4986934 | 152201875 |
| ESR1 | 6 | rs6557170 | 152203104 |
| ESR1 | 6 | rs11155820 | 152204210 |
| ESR1 | 6 | rs7761846 | 152212508 |
| ESR1 | 6 | rs1514347 | 152229445 |
| ESR1 | 6 | rs2347867 | 152229850 |
| ESR1 | 6 | rs6557171 | 152234593 |
| ESR1 | 6 | rs9397072 | 152239321 |
| ESR1 | 6 | rs988328 | 152241150 |
| ESR1 | 6 | rs6912184 | 152260206 |
| ESR1 | 6 | rs4583998 | 152260668 |
| ESR1 | 6 | rs4262200 | 152261004 |
| ESR1 | 6 | rs1801132 | 152265522 |
| ESR1 | 6 | rs3020410 | 152266377 |
| ESR1 | 6 | rs3003917 | 152266468 |
| ESR1 | 6 | rs3020424 | 152268483 |
| ESR1 | 6 | rs3020314 | 152270672 |
| ESR1 | 6 | rs3020391 | 152276923 |
| ESR1 | 6 | rs3003921 | 152279514 |
| ESR1 | 6 | rs3020401 | 152283044 |
| ESR1 | 6 | rs985191 | 152283458 |
| ESR1 | 6 | rs3003925 | 152284458 |
| ESR1 | 6 | rs2982688 | 152285122 |
| ESR1 | 6 | rs985695 | 152286705 |
| ESR1 | 6 | rs2347869 | 152287295 |
| ESR1 | 6 | rs2347871 | 152292660 |
| ESR1 | 6 | rs2347872 | 152292672 |
| ESR1 | 6 | rs3020325 | 152293905 |
| ESR1 | 6 | rs2982683 | 152298435 |
| ESR1 | 6 | rs726281 | 152302578 |
| ESR1 | 6 | rs728524 | 152303437 |
| ESR1 | 6 | rs9397463 | 152304328 |
| ESR1 | 6 | rs926777 | 152305047 |
| ESR1 | 6 | rs2982684 | 152306204 |
| ESR1 | 6 | rs9371236 | 152306346 |
| ESR1 | 6 | rs3020407 | 152307261 |
| ESR1 | 6 | rs2144025 | 152307706 |
| ESR1 | 6 | rs7743290 | 152309132 |
| ESR1 | 6 | rs9340944 | 152313718 |
| ESR1 | 6 | rs722208 | 152322885 |
| ESR1 | 6 | rs13216134 | 152328484 |
| ESR1 | 6 | rs1569788 | 152328616 |
| ESR1 | 6 | rs9340958 | 152330673 |
| ESR1 | 6 | rs9340969 | 152332130 |
| ESR1 | 6 | rs13203975 | 152333104 |
| ESR1 | 6 | rs9340978 | 152333945 |
| ESR1 | 6 | rs3020418 | 152345162 |
| ESR1 | 6 | rs9478265 | 152348901 |
| ESR1 | 6 | rs6941835 | 152356270 |
| ESR1 | 6 | rs2982712 | 152358179 |
| ESR1 | 6 | rs3020434 | 152358940 |
| ESR1 | 6 | rs3020365 | 152367993 |
| ESR1 | 6 | rs3020366 | 152368758 |
| ESR1 | 6 | rs3020368 | 152371190 |
| ESR1 | 6 | rs9383962 | 152375362 |
| ESR1 | 6 | rs6932864 | 152376475 |
| ESR1 | 6 | rs6913408 | 152378112 |
| ESR1 | 6 | rs2273206 | 152382311 |
| ESR1 | 6 | rs2273207 | 152382325 |
| ESR1 | 6 | rs2207396 | 152382382 |
| ESR1 | 6 | rs3778082 | 152387664 |
| ESR1 | 6 | rs3020375 | 152389968 |
| ESR1 | 6 | rs12199102 | 152392561 |
| ESR1 | 6 | rs9479190 | 152393112 |
| ESR1 | 6 | rs3822990 | 152405965 |
| ESR1 | 6 | rs3020382 | 152412137 |
| ESR1 | 6 | rs2982900 | 152414992 |
| ESR1 | 6 | rs9341052 | 152416625 |
| ESR1 | 6 | rs3778099 | 152418575 |
| ESR1 | 6 | rs2228480 | 152420095 |
| ESR1 | 6 | rs3798577 | 152421130 |
| ESR1 | 6 | rs3798758 | 152421854 |
| ESR1 | 6 | rs2747648 | 152422335 |
| ESR1 | 6 | rs9341077 | 152423128 |
| ESR1 | 6 | rs2813544 | 152425582 |
| ESR1 | 6 | rs910416 | 152432902 |
| ESR2 | 14 | rs45453691 | 64685212 |
| ESR2 | 14 | rs35648226 | 64686125 |
| ESR2 | 14 | rs8020646 | 64691320 |
| ESR2 | 14 | rs1152583 | 64692377 |
| ESR2 | 14 | rs1048315 | 64692465 |
| ESR2 | 14 | rs1152582 | 64692630 |
| ESR2 | 14 | rs7229 | 64692825 |
| ESR2 | 14 | rs2772163 | 64693385 |
| ESR2 | 14 | rs1255998 | 64693871 |
| ESR2 | 14 | rs8018687 | 64694082 |
| ESR2 | 14 | rs1256066 | 64698891 |
| ESR2 | 14 | rs8006145 | 64699450 |
| ESR2 | 14 | rs4986938 | 64699816 |
| ESR2 | 14 | kgp11395479 | 64700045 |
| ESR2 | 14 | rs1256064 | 64700739 |
| ESR2 | 14 | rs10144225 | 64704994 |
| ESR2 | 14 | rs8017441 | 64715794 |
| ESR2 | 14 | rs4365213 | 64720264 |
| ESR2 | 14 | rs1256049 | 64724051 |
| ESR2 | 14 | rs8003490 | 64725369 |
| ESR2 | 14 | rs1273196 | 64739505 |
| ESR2 | 14 | rs10143616 | 64740175 |
| ESR2 | 14 | rs10136955 | 64745263 |
| ESR2 | 14 | rs1256031 | 64746179 |
| ESR2 | 14 | rs1256030 | 64747170 |
| ESR2 | 14 | rs3783736 | 64751372 |
| ESR2 | 14 | rs1887994 | 64760611 |
| ESR2 | 14 | rs1271572 | 64761917 |
| ESR2 | 14 | rs2987983 | 64763653 |
| ESR2 | 14 | rs2978381 | 64766652 |
| ESR2 | 14 | rs3020450 | 64768302 |
| ESR2 | 14 | rs10137185 | 64775776 |
| ESR2 | 14 | rs3020443 | 64792340 |
| ESR2 | 14 | rs17101774 | 64793581 |
| ESR2 | 14 | rs17226088 | 64804157 |
| ESR2 | 14 | rs1256114 | 64810005 |
| CYP19A1 | 15 | rs9972359 | 51491854 |
| CYP19A1 | 15 | rs16964189 | 51494237 |
| CYP19A1 | 15 | rs4775932 | 51498539 |
| CYP19A1 | 15 | rs2255192 | 51500835 |
| CYP19A1 | 15 | rs12148604 | 51501404 |
| CYP19A1 | 15 | rs4646 | 51502844 |
| CYP19A1 | 15 | rs10046 | 51502986 |
| CYP19A1 | 15 | rs17601241 | 51507874 |
| CYP19A1 | 15 | rs700519 | 51507968 |
| CYP19A1 | 15 | rs9806371 | 51511771 |
| CYP19A1 | 15 | rs6493489 | 51514203 |
| CYP19A1 | 15 | rs28757184 | 51514572 |
| CYP19A1 | 15 | rs16964201 | 51515351 |
| CYP19A1 | 15 | rs8025374 | 51518370 |
| CYP19A1 | 15 | rs2899473 | 51519073 |
| CYP19A1 | 15 | rs4775935 | 51519276 |
| CYP19A1 | 15 | rs1065778 | 51520206 |
| CYP19A1 | 15 | rs2414095 | 51524292 |
| CYP19A1 | 15 | rs12592697 | 51525173 |
| CYP19A1 | 15 | rs9944225 | 51528080 |
| CYP19A1 | 15 | rs700518 | 51529112 |
| CYP19A1 | 15 | rs17703883 | 51530097 |
| CYP19A1 | 15 | rs28757162 | 51535885 |
| CYP19A1 | 15 | rs10459592 | 51536141 |
| CYP19A1 | 15 | rs10851498 | 51537012 |
| CYP19A1 | 15 | rs12591359 | 51539368 |
| CYP19A1 | 15 | rs11632036 | 51542162 |
| CYP19A1 | 15 | rs12911554 | 51542757 |
| CYP19A1 | 15 | rs16964220 | 51543382 |
| CYP19A1 | 15 | rs28757158 | 51544970 |
| CYP19A1 | 15 | rs12907866 | 51545454 |
| CYP19A1 | 15 | rs7172156 | 51546298 |
| CYP19A1 | 15 | rs2008691 | 51548310 |
| CYP19A1 | 15 | rs2414099 | 51548782 |
| CYP19A1 | 15 | rs1008805 | 51549599 |
| CYP19A1 | 15 | rs28757152 | 51550831 |
| CYP19A1 | 15 | rs749292 | 51558731 |
| CYP19A1 | 15 | rs2305707 | 51569410 |
| CYP19A1 | 15 | rs28757128 | 51569947 |
| CYP19A1 | 15 | rs8029807 | 51572037 |
| CYP19A1 | 15 | rs7167343 | 51578594 |
| CYP19A1 | 15 | rs936306 | 51579598 |
| CYP19A1 | 15 | rs9302160 | 51590011 |
| CYP19A1 | 15 | rs2470152 | 51594972 |
| CYP19A1 | 15 | rs10519302 | 51599683 |
| CYP19A1 | 15 | rs3751592 | 51606578 |
| CYP19A1 | 15 | rs3751591 | 51606710 |
| CYP19A1 | 15 | rs2470151 | 51607069 |
| CYP19A1 | 15 | rs1004984 | 51613529 |
| CYP19A1 | 15 | rs4774585 | 51616480 |
| CYP19A1 | 15 | rs2445762 | 51617708 |
| CYP19A1 | 15 | rs2470144 | 51621725 |
| CYP19A1 | 15 | rs7174997 | 51622128 |
| CYP19A1 | 15 | rs1870049 | 51625223 |
| CYP19A1 | 15 | rs6493497 | 51630835 |
| CYP19A1 | 15 | rs11631118 | 51633396 |
| CYP19A1 | 15 | rs12441859 | 51637250 |
| CYP19A1 | 15 | rs12595379 | 51639772 |
| CYP19A1 | 15 | rs2124872 | 51639821 |
| CYP19A1 | 15 | rs2445771 | 51640359 |
| OXT | 20 | rs6084253 | 3046062 |
| OXT | 20 | rs6084258 | 3048495 |
| OXT | 20 | rs11697250 | 3048537 |
| OXT | 20 | rs877172 | 3049890 |
| OXT | 20 | rs3761248 | 3050393 |
| OXT | 20 | rs2770378 | 3053514 |
| OXT | 20 | rs4813627 | 3055513 |
| OXT | 20 | rs2740201 | 3056686 |
| OXT | 20 | rs2740185 | 3061436 |
| OXT | 20 | rs2770381 | 3061986 |
| OXTR | 3 | rs237875 | 8782406 |
| OXTR | 3 | rs237877 | 8782887 |
| OXTR | 3 | rs6777088 | 8786487 |
| OXTR | 3 | rs13087941 | 8787220 |
| OXTR | 3 | rs13093809 | 8788096 |
| OXTR | 3 | rs11476 | 8788198 |
| OXTR | 3 | rs7629329 | 8788336 |
| OXTR | 3 | rs2324728 | 8792728 |
| OXTR | 3 | rs237884 | 8793585 |
| OXTR | 3 | rs6770632 | 8793724 |
| OXTR | 3 | rs1042778 | 8794545 |
| OXTR | 3 | rs237885 | 8795543 |
| OXTR | 3 | rs11706648 | 8796547 |
| OXTR | 3 | rs237887 | 8797042 |
| OXTR | 3 | rs2268490 | 8797085 |
| OXTR | 3 | rs237888 | 8797095 |
| OXTR | 3 | rs918316 | 8798181 |
| OXTR | 3 | rs4686301 | 8798586 |
| OXTR | 3 | rs2268491 | 8800398 |
| OXTR | 3 | rs2254298 | 8802228 |
| OXTR | 3 | rs237889 | 8802483 |
| OXTR | 3 | rs11131149 | 8802851 |
| OXTR | 3 | rs237895 | 8807423 |
| OXTR | 3 | rs2268495 | 8807535 |
| OXTR | 3 | rs237897 | 8808285 |
| OXTR | 3 | rs237899 | 8808515 |
| OXTR | 3 | rs237902 | 8809184 |
| OXTR | 3 | kgp3933398 | 8809222 |
| OXTR | 3 | rs237911 | 8810008 |
| OXTR | 3 | rs2301261 | 8810896 |
| OXTR | 3 | rs6777726 | 8813494 |
| OXTR | 3 | rs180789 | 8813927 |
| OXTR | 3 | rs6443206 | 8820075 |
| OXTR | 3 | rs75775 | 8820732 |
| OXTR | 3 | rs9860869 | 8820740 |
| CD38 | 4 | rs11945730 | 15773008 |
| CD38 | 4 | rs16892407 | 15773936 |
| CD38 | 4 | rs6831585 | 15776181 |
| CD38 | 4 | rs6449181 | 15779712 |
| CD38 | 4 | rs3756243 | 15784598 |
| CD38 | 4 | rs4580644 | 15785201 |
| CD38 | 4 | rs3756242 | 15786002 |
| CD38 | 4 | rs10004415 | 15788885 |
| CD38 | 4 | rs7666953 | 15791464 |
| CD38 | 4 | rs10016073 | 15797704 |
| CD38 | 4 | rs12644506 | 15799878 |
| CD38 | 4 | rs6449191 | 15800268 |
| CD38 | 4 | rs16892418 | 15810779 |
| CD38 | 4 | rs6836946 | 15813607 |
| CD38 | 4 | rs7655635 | 15814621 |
| CD38 | 4 | rs6449195 | 15814814 |
| CD38 | 4 | rs6449197 | 15814922 |
| CD38 | 4 | rs3796868 | 15818391 |
| CD38 | 4 | rs3796867 | 15818548 |
| CD38 | 4 | rs3796866 | 15835365 |
| CD38 | 4 | rs1800051 | 15835844 |
| CD38 | 4 | rs3796863 | 15849986 |
| CD38 | 4 | rs1803404 | 15850330 |
| CD38 | 4 | rs1130169 | 15850685 |
| CD38 | 4 | rs13137313 | 15851968 |
| CD38 | 4 | rs17476066 | 15852104 |
| CD38 | 4 | rs3733593 | 15854725 |
| CD38 | 4 | rs13136270 | 15856799 |
| CD38 | 4 | rs10001128 | 15858862 |
