# Supplementary material for: Polymorphisms in Genes of Relevance for Oestrogen and Oxytocin Pathways and Risk of Barrett’s Oesophagus and Oesophageal Adenocarcinoma: A Pooled Analysis from the BEACON Consortium
Source: PLoS One. 2015 Sep 25;10(9):e0138738. doi: 10.1371/journal.pone.0138738 (PMC4583498; doi:10.1371/journal.pone.0138738)
Supplement: S3 Table — (DOCX) [file pone.0138738.s004.docx]

**Supporting Information**

**S4 Table.** Single nucleotide polymorphisms (SNPs) results in the total sample for the oestrogen receptor alpha gene (*ESR1*) and risk of Barrett’s oesophagus (BO), oesophageal adenocarcinoma (OAC), and these conditions combined (BO+OAC). P-values in bold are nominally significant.

|  | | | |  | | | **BO** | | |  | | **OAC** | | |  | | **BO+OAC** | |  |  |  |
| --- | --- | --- | --- | --- | --- | --- | --- | --- | --- | --- | --- | --- | --- | --- | --- | --- | --- | --- | --- | --- | --- |
| **CHR^1^** | **SNP** | **BP^2^** | **A1^3^** | |  | **OR^4^** | | **P^5^** |  | | **OR^4^** | | **P^5^** |  | | **OR^4^** | | **P^5^** |  | **Position** | |
| 6 | rs6557164 | 152002932 | A | |  | 1.01 | | 0.83 |  | | 0.98 | | 0.78 |  | | 1.00 | | 0.95 |  |  | |
| 6 | rs851995 | 152005534 | G | |  | 0.98 | | 0.60 |  | | 0.93 | | 0.16 |  | | 0.96 | | 0.35 |  |  | |
| 6 | rs851993 | 152006011 | G | |  | 1.04 | | 0.43 |  | | 1.00 | | 0.98 |  | | 1.03 | | 0.51 |  |  | |
| 6 | rs9383939 | 152006178 | A | |  | 0.82 | | **0.01** |  | | 0.82 | | **0.04** |  | | 0.82 | | **0.005** |  |  | |
| 6 | rs851991 | 152006581 | A | |  | 1.06 | | 0.17 |  | | 1.05 | | 0.32 |  | | 1.05 | | 0.18 |  |  | |
| 6 | rs851987 | 152007889 | A | |  | 0.98 | | 0.60 |  | | 1.04 | | 0.49 |  | | 1.00 | | 0.93 |  |  | |
| 6 | kgp10311820 | 152008442 | A | |  | 1.04 | | 0.77 |  | | 1.00 | | 0.97 |  | | 1.02 | | 0.85 |  |  | |
| 6 | rs3020331 | 152008780 | A | |  | 1.04 | | 0.36 |  | | 1.05 | | 0.37 |  | | 1.04 | | 0.35 |  |  | |
| 6 | kgp3926446 | 152008924 | A | |  | 1.04 | | 0.39 |  | | 1.06 | | 0.27 |  | | 1.04 | | 0.33 |  |  | |
| 6 | kgp6713052 | 152009427 | A | |  | 0.99 | | 0.91 |  | | 1.00 | | 0.97 |  | | 0.99 | | 0.93 |  |  | |
| 6 | rs2941740 | 152009638 | G | |  | 1.05 | | 0.29 |  | | 1.06 | | 0.23 |  | | 1.05 | | 0.25 |  |  | |
| 6 | rs3020333 | 152010254 | G | |  | 1.05 | | 0.30 |  | | 1.06 | | 0.21 |  | | 1.05 | | 0.25 |  |  | |
| 6 | kgp6718446 | 152010377 | A | |  | 0.91 | | 0.44 |  | | 0.90 | | 0.49 |  | | 0.92 | | 0.46 |  |  | |
| 6 | kgp12313848 | 152010534 | G | |  | 1.05 | | 0.30 |  | | 1.06 | | 0.22 |  | | 1.05 | | 0.25 |  |  | |
| 6 | kgp2093333 | 152010561 | A | |  | 1.05 | | 0.30 |  | | 1.06 | | 0.21 |  | | 1.05 | | 0.25 |  |  | |
| 6 | kgp11248855 | 152012739 | T | |  | 1.05 | | 0.26 |  | | 1.06 | | 0.20 |  | | 1.05 | | 0.22 |  |  | |
| 6 | rs3020334 | 152012956 | G | |  | 1.05 | | 0.26 |  | | 1.07 | | 0.20 |  | | 1.05 | | 0.21 |  |  | |
| 6 | kgp737782 | 152012988 | G | |  | 1.05 | | 0.26 |  | | 1.07 | | 0.20 |  | | 1.05 | | 0.21 |  |  | |
| 6 | kgp1921179 | 152013748 | A | |  | 1.05 | | 0.24 |  | | 1.06 | | 0.21 |  | | 1.05 | | 0.20 |  |  | |
| 6 | kgp9736427 | 152013760 | A | |  | 1.05 | | 0.25 |  | | 1.06 | | 0.21 |  | | 1.05 | | 0.21 |  |  | |
| 6 | kgp11394755 | 152016803 | A | |  | 1.05 | | 0.29 |  | | 1.07 | | 0.16 |  | | 1.05 | | 0.21 |  |  | |
| 6 | rs1293936 | 152017691 | C | |  | 1.04 | | 0.34 |  | | 1.08 | | 0.14 |  | | 1.05 | | 0.22 |  |  | |
| 6 | kgp2922863 | 152018139 | G | |  | 0.97 | | 0.82 |  | | 0.99 | | 0.94 |  | | 0.98 | | 0.85 |  |  | |
| 6 | kgp3955218 | 152018498 | G | |  | 0.97 | | 0.82 |  | | 0.99 | | 0.94 |  | | 0.98 | | 0.85 |  |  | |
| 6 | kgp705069 | 152020390 | C | |  | 1.04 | | 0.32 |  | | 1.07 | | 0.18 |  | | 1.05 | | 0.24 |  |  | |
| 6 | kgp1417894 | 152021049 | G | |  | 0.94 | | 0.58 |  | | 0.97 | | 0.83 |  | | 0.95 | | 0.58 |  |  | |
| 6 | kgp9300867 | 152023191 | A | |  | 1.04 | | 0.35 |  | | 1.06 | | 0.23 |  | | 1.04 | | 0.28 |  |  | |
| 6 | kgp10100871 | 152024178 | A | |  | 0.99 | | 0.91 |  | | 1.00 | | 0.98 |  | | 0.99 | | 0.93 |  |  | |
| 6 | rs851983 | 152024415 | G | |  | 1.04 | | 0.33 |  | | 1.06 | | 0.22 |  | | 1.05 | | 0.26 |  |  | |
| 6 | rs851982 | 152024985 | G | |  | 1.04 | | 0.32 |  | | 1.06 | | 0.21 |  | | 1.05 | | 0.25 |  |  | |
| 6 | kgp17127196 | 152027012 | C | |  | 0.86 | | 0.41 |  | | 0.74 | | 0.16 |  | | 0.82 | | 0.24 |  |  | |
| 6 | kgp2475865 | 152027074 | A | |  | 1.03 | | 0.56 |  | | 1.13 | | **0.02** |  | | 1.06 | | 0.16 |  |  | |
| 6 | kgp5788762 | 152027955 | G | |  | 1.03 | | 0.57 |  | | 1.14 | | **0.02** |  | | 1.06 | | 0.16 |  |  | |
| 6 | kgp6899289 | 152028755 | G | |  | 0.98 | | 0.88 |  | | 0.99 | | 0.94 |  | | 0.99 | | 0.89 |  |  | |
| 6 | rs851978 | 152029556 | A | |  | 1.04 | | 0.43 |  | | 1.12 | | **0.04** |  | | 1.06 | | 0.15 |  |  | |
| 6 | kgp5232249 | 152031303 | A | |  | 1.04 | | 0.40 |  | | 1.13 | | **0.03** |  | | 1.07 | | 0.13 |  |  | |
| 6 | kgp9690084 | 152031496 | A | |  | 1.05 | | 0.71 |  | | 1.28 | | 0.08 |  | | 1.12 | | 0.34 |  |  | |
| 6 | kgp11951474 | 152039385 | C | |  | 0.99 | | 0.92 |  | | 1.00 | | 0.98 |  | | 0.99 | | 0.94 |  |  | |
| 6 | kgp9635185 | 152039444 | G | |  | 0.99 | | 0.92 |  | | 1.00 | | 0.98 |  | | 0.99 | | 0.94 |  |  | |
| 6 | kgp9033598 | 152039889 | A | |  | 1.25 | | 0.13 |  | | 1.32 | | 0.09 |  | | 1.30 | | 0.05 |  |  | |
| 6 | kgp5012159 | 152039964 | G | |  | 1.04 | | 0.44 |  | | 1.12 | | **0.04** |  | | 1.06 | | 0.16 |  |  | |
| 6 | kgp3691071 | 152040125 | G | |  | 1.04 | | 0.42 |  | | 1.12 | | **0.04** |  | | 1.07 | | 0.15 |  |  | |
| 6 | kgp10208621 | 152040257 | G | |  | 0.98 | | 0.87 |  | | 0.99 | | 0.94 |  | | 0.98 | | 0.88 |  |  | |
| 6 | rs12525163 | 152040291 | G | |  | 1.04 | | 0.45 |  | | 1.12 | | **0.03** |  | | 1.06 | | 0.15 |  |  | |
| 6 | kgp9534209 | 152040615 | A | |  | 1.04 | | 0.43 |  | | 1.12 | | **0.03** |  | | 1.07 | | 0.14 |  |  | |
| 6 | rs10484921 | 152042260 | A | |  | 1.04 | | 0.44 |  | | 1.13 | | **0.03** |  | | 1.07 | | 0.15 |  | **upstream** | |
| 6 | kgp10486525 | 152042413 | T | |  | 1.03 | | 0.48 |  | | 1.11 | | 0.05 |  | | 1.06 | | 0.18 |  |  | |
| 6 | kgp1144405 | 152042502 | C | |  | 1.04 | | 0.45 |  | | 1.12 | | **0.04** |  | | 1.06 | | 0.16 |  |  | |
| 6 | kgp11513921 | 152043290 | G | |  | 1.04 | | 0.46 |  | | 1.11 | | **0.04** |  | | 1.06 | | 0.17 |  |  | |
| 6 | rs1159327 | 152048022 | A | |  | 1.03 | | 0.56 |  | | 1.11 | | 0.06 |  | | 1.05 | | 0.23 |  |  | |
| 6 | kgp2380438 | 152048217 | G | |  | 1.00 | | 0.97 |  | | 0.98 | | 0.90 |  | | 0.99 | | 0.94 |  |  | |
| 6 | kgp6354401 | 152049501 | G | |  | 1.00 | | 0.97 |  | | 0.98 | | 0.91 |  | | 0.99 | | 0.94 |  |  | |
| 6 | kgp2855478 | 152051854 | A | |  | 1.07 | | 0.25 |  | | 1.10 | | 0.13 |  | | 1.08 | | 0.13 |  |  | |
| 6 | kgp6631336 | 152052043 | A | |  | 1.03 | | 0.50 |  | | 1.06 | | 0.32 |  | | 1.04 | | 0.37 |  |  | |
| 6 | kgp7957493 | 152052601 | G | |  | 0.95 | | 0.24 |  | | 0.94 | | 0.18 |  | | 0.95 | | 0.18 |  |  | |
| 6 | kgp11024871 | 152052652 | G | |  | 0.95 | | 0.24 |  | | 0.94 | | 0.17 |  | | 0.95 | | 0.17 |  |  | |
| 6 | rs3020343 | 152054363 | G | |  | 0.95 | | 0.22 |  | | 0.92 | | 0.10 |  | | 0.94 | | 0.13 |  |  | |
| 6 | rs12195741 | 152054374 | A | |  | 1.04 | | 0.40 |  | | 1.01 | | 0.89 |  | | 1.03 | | 0.54 |  |  | |
| 6 | kgp12208636 | 152055606 | A | |  | 0.95 | | 0.24 |  | | 0.92 | | 0.10 |  | | 0.94 | | 0.13 |  |  | |
| 6 | kgp6494875 | 152056081 | A | |  | 0.95 | | 0.26 |  | | 0.92 | | 0.09 |  | | 0.94 | | 0.13 |  |  | |
| 6 | kgp1691518 | 152056146 | A | |  | 0.95 | | 0.27 |  | | 0.92 | | 0.08 |  | | 0.94 | | 0.14 |  |  | |
| 6 | kgp1103762 | 152056368 | A | |  | 0.95 | | 0.22 |  | | 0.92 | | 0.09 |  | | 0.94 | | 0.12 |  |  | |
| 6 | kgp5296648 | 152057408 | T | |  | 0.95 | | 0.25 |  | | 0.92 | | 0.08 |  | | 0.94 | | 0.13 |  |  | |
| 6 | rs3020348 | 152057914 | C | |  | 0.95 | | 0.25 |  | | 0.92 | | 0.11 |  | | 0.95 | | 0.14 |  |  | |
| 6 | kgp5641093 | 152058010 | A | |  | 0.95 | | 0.23 |  | | 0.92 | | 0.09 |  | | 0.94 | | 0.13 |  |  | |
| 6 | kgp8324978 | 152058844 | T | |  | 0.96 | | 0.30 |  | | 0.92 | | 0.11 |  | | 0.95 | | 0.16 |  |  | |
| 6 | rs2982552 | 152059563 | G | |  | 0.97 | | 0.46 |  | | 0.92 | | 0.09 |  | | 0.95 | | 0.21 |  |  | |
| 6 | kgp10613698 | 152059787 | G | |  | 0.99 | | 0.92 |  | | 0.97 | | 0.85 |  | | 0.98 | | 0.88 |  |  | |
| 6 | rs2982551 | 152061210 | C | |  | 0.97 | | 0.41 |  | | 0.92 | | 0.08 |  | | 0.95 | | 0.19 |  |  | |
| 6 | kgp3397459 | 152062530 | A | |  | 0.99 | | 0.92 |  | | 0.96 | | 0.77 |  | | 0.98 | | 0.84 |  |  | |
| 6 | kgp8113620 | 152063998 | A | |  | 0.98 | | 0.65 |  | | 0.93 | | 0.17 |  | | 0.97 | | 0.38 |  |  | |
| 6 | kgp2765867 | 152064199 | C | |  | 0.98 | | 0.64 |  | | 0.93 | | 0.15 |  | | 0.97 | | 0.36 |  |  | |
| 6 | kgp5408731 | 152064355 | G | |  | 0.98 | | 0.65 |  | | 0.93 | | 0.14 |  | | 0.97 | | 0.35 |  |  | |
| 6 | kgp3560501 | 152064454 | A | |  | 0.98 | | 0.66 |  | | 0.94 | | 0.17 |  | | 0.97 | | 0.38 |  |  | |
| 6 | kgp2861858 | 152064464 | G | |  | 0.98 | | 0.60 |  | | 0.93 | | 0.14 |  | | 0.96 | | 0.33 |  |  | |
| 6 | kgp3189538 | 152064487 | A | |  | 0.98 | | 0.64 |  | | 0.93 | | 0.15 |  | | 0.97 | | 0.36 |  |  | |
| 6 | rs3020306 | 152065886 | G | |  | 0.98 | | 0.60 |  | | 0.93 | | 0.16 |  | | 0.96 | | 0.33 |  |  | |
| 6 | rs1856057 | 152067869 | A | |  | 0.98 | | 0.66 |  | | 0.93 | | 0.17 |  | | 0.97 | | 0.37 |  |  | |
| 6 | rs1999805 | 152068364 | G | |  | 0.98 | | 0.66 |  | | 0.94 | | 0.18 |  | | 0.97 | | 0.38 |  |  | |
| 6 | kgp8502758 | 152068591 | G | |  | 0.99 | | 0.94 |  | | 0.96 | | 0.78 |  | | 0.98 | | 0.85 |  |  | |
| 6 | kgp4577083 | 152068685 | A | |  | 0.98 | | 0.66 |  | | 0.94 | | 0.18 |  | | 0.97 | | 0.38 |  |  | |
| 6 | kgp8802865 | 152068874 | G | |  | 0.98 | | 0.66 |  | | 0.94 | | 0.20 |  | | 0.97 | | 0.40 |  |  | |
| 6 | kgp8372881 | 152069791 | A | |  | 0.98 | | 0.66 |  | | 0.93 | | 0.15 |  | | 0.97 | | 0.36 |  |  | |
| 6 | kgp9144294 | 152069999 | A | |  | 0.98 | | 0.62 |  | | 0.93 | | 0.16 |  | | 0.96 | | 0.34 |  |  | |
| 6 | kgp11639523 | 152072718 | A | |  | 0.93 | | 0.38 |  | | 1.09 | | 0.36 |  | | 0.98 | | 0.76 |  |  | |
| 6 | kgp2911751 | 152075487 | G | |  | 0.98 | | 0.63 |  | | 0.94 | | 0.21 |  | | 0.97 | | 0.39 |  |  | |
| 6 | kgp3916874 | 152080039 | A | |  | 1.08 | | 0.47 |  | | 1.05 | | 0.70 |  | | 1.08 | | 0.40 |  |  | |
| 6 | kgp4245848 | 152081009 | A | |  | 1.01 | | 0.84 |  | | 0.93 | | 0.18 |  | | 0.99 | | 0.70 |  |  | |
| 6 | rs1336981 | 152082369 | G | |  | 0.99 | | 0.90 |  | | 0.93 | | 0.15 |  | | 0.97 | | 0.47 |  |  | |
| 6 | kgp5179039 | 152082646 | A | |  | 1.05 | | 0.75 |  | | 0.79 | | 0.23 |  | | 0.94 | | 0.70 |  |  | |
| 6 | kgp584372 | 152082973 | T | |  | 1.05 | | 0.76 |  | | 0.77 | | 0.18 |  | | 0.94 | | 0.65 |  |  | |
| 6 | rs9479109 | 152084593 | C | |  | 1.06 | | 0.74 |  | | 0.78 | | 0.21 |  | | 0.94 | | 0.69 |  |  | |
| 6 | kgp6294659 | 152084862 | A | |  | 1.01 | | 0.79 |  | | 0.91 | | 0.05 |  | | 0.97 | | 0.50 |  |  | |
| 6 | kgp8301700 | 152084973 | A | |  | 1.05 | | 0.25 |  | | 0.97 | | 0.59 |  | | 1.03 | | 0.52 |  |  | |
| 6 | rs1890010 | 152085275 | G | |  | 1.03 | | 0.52 |  | | 0.95 | | 0.38 |  | | 1.01 | | 0.91 |  |  | |
| 6 | kgp7859664 | 152087111 | C | |  | 1.01 | | 0.95 |  | | 0.98 | | 0.91 |  | | 1.00 | | 0.97 |  |  | |
| 6 | kgp7324582 | 152087318 | A | |  | 1.02 | | 0.89 |  | | 1.00 | | 0.99 |  | | 1.01 | | 0.96 |  |  | |
| 6 | kgp8702359 | 152088155 | A | |  | 0.91 | | 0.11 |  | | 0.89 | | 0.11 |  | | 0.90 | | 0.05 |  |  | |
| 6 | rs2485209 | 152089768 | C | |  | 0.94 | | 0.17 |  | | 0.93 | | 0.16 |  | | 0.94 | | 0.09 |  |  | |
| 6 | kgp10166489 | 152090024 | A | |  | 0.94 | | 0.24 |  | | 0.89 | | 0.05 |  | | 0.92 | | 0.07 |  |  | |
| 6 | rs6939257 | 152090045 | G | |  | 0.94 | | 0.23 |  | | 0.89 | | 0.05 |  | | 0.92 | | 0.07 |  |  | |
| 6 | rs6939683 | 152090324 | G | |  | 0.91 | | 0.11 |  | | 0.90 | | 0.12 |  | | 0.90 | | 0.06 |  |  | |
| 6 | kgp7561639 | 152090535 | A | |  | 0.91 | | 0.12 |  | | 0.90 | | 0.13 |  | | 0.91 | | 0.06 |  |  | |
| 6 | rs2504063 | 152090707 | A | |  | 1.00 | | 0.94 |  | | 0.90 | | **0.03** |  | | 0.96 | | 0.31 |  |  | |
| 6 | rs9371553 | 152091428 | C | |  | 1.05 | | 0.76 |  | | 0.73 | | 0.11 |  | | 0.93 | | 0.59 |  |  | |
| 6 | kgp4921988 | 152092638 | T | |  | 1.06 | | 0.16 |  | | 1.10 | | 0.06 |  | | 1.08 | | 0.06 |  |  | |
| 6 | rs4870053 | 152092749 | A | |  | 0.96 | | 0.43 |  | | 0.91 | | 0.09 |  | | 0.94 | | 0.19 |  |  | |
| 6 | kgp1077733 | 152094838 | G | |  | 0.92 | | 0.13 |  | | 0.90 | | 0.11 |  | | 0.91 | | 0.06 |  |  | |
| 6 | kgp11619301 | 152095043 | A | |  | 0.99 | | 0.95 |  | | 0.98 | | 0.86 |  | | 0.98 | | 0.88 |  |  | |
| 6 | rs2504065 | 152095167 | A | |  | 1.00 | | 0.91 |  | | 1.04 | | 0.47 |  | | 1.01 | | 0.78 |  |  | |
| 6 | rs2248586 | 152095332 | G | |  | 1.02 | | 0.70 |  | | 1.02 | | 0.69 |  | | 1.02 | | 0.71 |  |  | |
| 6 | kgp9819860 | 152095694 | G | |  | 0.91 | | 0.08 |  | | 0.90 | | 0.10 |  | | 0.90 | | 0.04 |  |  | |
| 6 | kgp7268606 | 152095829 | G | |  | 1.00 | | 0.92 |  | | 1.05 | | 0.27 |  | | 1.02 | | 0.64 |  |  | |
| 6 | rs2504067 | 152095987 | A | |  | 0.99 | | 0.81 |  | | 1.08 | | 0.12 |  | | 1.02 | | 0.57 |  |  | |
| 6 | rs17828471 | 152097405 | G | |  | 0.95 | | 0.45 |  | | 1.09 | | 0.21 |  | | 1.00 | | 0.98 |  |  | |
| 6 | kgp9235060 | 152098571 | A | |  | 1.00 | | 0.97 |  | | 1.17 | | 0.06 |  | | 1.06 | | 0.41 |  |  | |
| 6 | kgp6725383 | 152098687 | G | |  | 1.00 | | 0.97 |  | | 1.16 | | 0.08 |  | | 1.05 | | 0.44 |  |  | |
| 6 | kgp1450269 | 152101823 | A | |  | 0.99 | | 0.95 |  | | 1.16 | | 0.07 |  | | 1.05 | | 0.45 |  |  | |
| 6 | rs528529 | 152102939 | C | |  | 0.99 | | 0.77 |  | | 0.96 | | 0.40 |  | | 0.98 | | 0.55 |  |  | |
| 6 | rs1285057 | 152103792 | A | |  | 1.01 | | 0.86 |  | | 0.96 | | 0.37 |  | | 0.99 | | 0.79 |  |  | |
| 6 | rs543650 | 152110943 | A | |  | 1.10 | | **0.03** |  | | 0.93 | | 0.15 |  | | 1.03 | | 0.40 |  |  | |
| 6 | rs17081685 | 152116655 | G | |  | 0.94 | | 0.45 |  | | 0.99 | | 0.95 |  | | 0.96 | | 0.59 |  |  | |
| 6 | rs2881766 | 152119119 | C | |  | 0.99 | | 0.80 |  | | 1.08 | | 0.18 |  | | 1.02 | | 0.64 |  |  | |
| 6 | rs11964281 | 152121442 | A | |  | 1.01 | | 0.85 |  | | 1.15 | | 0.09 |  | | 1.06 | | 0.36 |  |  | |
| 6 | rs488133 | 152125444 | A | |  | 1.08 | | 0.09 |  | | 0.94 | | 0.23 |  | | 1.03 | | 0.54 |  |  | |
| 6 | rs2071454 | 152126824 | C | |  | 1.05 | | 0.49 |  | | 1.18 | | **0.03** |  | | 1.10 | | 0.13 |  |  | |
| 6 | rs2077647 | 152129077 | G | |  | 0.97 | | 0.53 |  | | 1.09 | | 0.08 |  | | 1.02 | | 0.67 |  |  | |
| 6 | rs532010 | 152130918 | G | |  | 0.97 | | 0.42 |  | | 1.05 | | 0.38 |  | | 1.00 | | 0.93 |  |  | |
| 6 | rs3853248 | 152139886 | G | |  | 1.02 | | 0.78 |  | | 1.13 | | 0.08 |  | | 1.06 | | 0.31 |  |  | |
| 6 | rs9371557 | 152140209 | G | |  | 0.76 | | 0.17 |  | | 1.02 | | 0.93 |  | | 0.86 | | 0.37 |  |  | |
| 6 | rs3844509 | 152140464 | G | |  | 1.02 | | 0.77 |  | | 1.13 | | 0.08 |  | | 1.06 | | 0.31 |  |  | |
| 6 | rs7759411 | 152148870 | A | |  | 1.33 | | 0.14 |  | | 1.59 | | **0.03** |  | | 1.45 | | 0.04 |  |  | |
| 6 | rs11969288 | 152149200 | A | |  | 0.85 | | 0.32 |  | | 0.84 | | 0.36 |  | | 0.84 | | 0.21 |  |  | |
| 6 | rs11155813 | 152149435 | G | |  | 1.05 | | 0.45 |  | | 1.18 | | **0.03** |  | | 1.10 | | 0.11 |  |  | |
| 6 | rs7761133 | 152151863 | G | |  | 0.98 | | 0.79 |  | | 1.10 | | 0.16 |  | | 1.03 | | 0.62 |  |  | |
| 6 | rs6909023 | 152153697 | A | |  | 0.88 | | 0.27 |  | | 0.94 | | 0.64 |  | | 0.91 | | 0.35 |  |  | |
| 6 | rs827423 | 152156197 | G | |  | 0.97 | | 0.53 |  | | 0.93 | | 0.11 |  | | 0.99 | | 0.74 |  |  | |
| 6 | rs827421 | 152157122 | G | |  | 0.97 | | 0.54 |  | | 0.93 | | 0.16 |  | | 0.99 | | 0.79 |  |  | |
| 6 | rs6902771 | 152157881 | A | |  | 0.98 | | 0.62 |  | | 1.09 | | 0.08 |  | | 1.02 | | 0.66 |  |  | |
| 6 | rs9322331 | 152162317 | A | |  | 0.96 | | 0.36 |  | | 1.04 | | 0.51 |  | | 0.99 | | 0.75 |  |  | |
| 6 | rs2234693 | 152163335 | G | |  | 0.97 | | 0.46 |  | | 1.07 | | 0.15 |  | | 1.01 | | 0.89 |  |  | |
| 6 | rs827419 | 152177663 | C | |  | 1.02 | | 0.67 |  | | 1.12 | | **0.02** |  | | 1.06 | | 0.16 |  |  | |
| 6 | rs1643821 | 152183551 | A | |  | 1.02 | | 0.60 |  | | 1.12 | | **0.02** |  | | 1.06 | | 0.15 |  |  | |
| 6 | rs11155818 | 152184130 | A | |  | 1.15 | | 0.44 |  | | 1.17 | | 0.46 |  | | 1.19 | | 0.31 |  |  | |
| 6 | rs1709183 | 152193996 | G | |  | 0.91 | | 0.05 |  | | 0.90 | | 0.06 |  | | 0.91 | | 0.03 |  |  | |
| 6 | rs11155819 | 152199359 | G | |  | 0.96 | | 0.38 |  | | 1.06 | | 0.26 |  | | 1.00 | | 0.90 |  |  | |
| 6 | rs9322335 | 152200129 | A | |  | 0.96 | | 0.37 |  | | 0.92 | | 0.16 |  | | 0.94 | | 0.19 |  |  | |
| 6 | rs9322336 | 152200430 | G | |  | 0.95 | | 0.28 |  | | 0.88 | | **0.03** |  | | 0.92 | | 0.08 |  |  | |
| 6 | rs4986934 | 152201875 | A | |  | 1.19 | | 0.10 |  | | 0.86 | | 0.26 |  | | 1.05 | | 0.58 |  |  | |
| 6 | rs6557170 | 152203104 | A | |  | 0.89 | | **0.02** |  | | 0.91 | | 0.12 |  | | 0.90 | | **0.02** |  | **intron 3** | |
| 6 | rs11155820 | 152204210 | G | |  | 1.08 | | 0.11 |  | | 1.11 | | **0.04** |  | | 1.09 | | **0.04** |  |  | |
| 6 | rs7761846 | 152212508 | G | |  | 0.90 | | 0.13 |  | | 0.83 | | **0.03** |  | | 0.87 | | **0.04** |  |  | |
| 6 | rs1514347 | 152229445 | A | |  | 0.89 | | **0.02** |  | | 0.91 | | 0.12 |  | | 0.90 | | **0.02** |  |  | |
| 6 | rs2347867 | 152229850 | G | |  | 0.87 | | **0.002** |  | | 0.88 | | **0.01** |  | | 0.87 | | **0.0006** |  |  | |
| 6 | rs6557171 | 152234593 | A | |  | 0.87 | | **0.002** |  | | 0.86 | | **0.004** |  | | 0.86 | | **0.0004** |  |  | |
| 6 | rs9397072 | 152239321 | A | |  | 0.96 | | 0.74 |  | | 1.05 | | 0.74 |  | | 0.99 | | 0.94 |  |  | |
| 6 | rs988328 | 152241150 | G | |  | 0.87 | | **0.02** |  | | 0.94 | | 0.33 |  | | 0.90 | | 0.05 |  |  | |
| 6 | rs6912184 | 152260206 | G | |  | 0.91 | | 0.06 |  | | 0.91 | | 0.10 |  | | 0.91 | | **0.04** |  |  | |
| 6 | rs4583998 | 152260668 | A | |  | 0.88 | | **0.006** |  | | 0.86 | | **0.005** |  | | 0.88 | | **0.001** |  | **intron 3** | |
| 6 | rs4262200 | 152261004 | G | |  | 0.91 | | 0.06 |  | | 0.91 | | 0.10 |  | | 0.91 | | **0.04** |  |  | |
| 6 | rs1801132 | 152265522 | C | |  | 0.91 | | 0.05 |  | | 0.90 | | 0.07 |  | | 0.91 | | **0.03** |  |  | |
| 6 | rs3020410 | 152266377 | A | |  | 0.96 | | 0.49 |  | | 0.89 | | 0.09 |  | | 0.93 | | 0.18 |  |  | |
| 6 | rs3003917 | 152266468 | G | |  | 0.88 | | **0.02** |  | | 0.90 | | 0.10 |  | | 0.89 | | **0.02** |  |  | |
| 6 | rs3020424 | 152268483 | A | |  | 0.94 | | 0.18 |  | | 0.90 | | **0.04** |  | | 0.93 | | 0.07 |  |  | |
| 6 | rs3020314 | 152270672 | G | |  | 0.93 | | 0.10 |  | | 0.91 | | 0.05 |  | | 0.92 | | 0.05 |  | **intron 4** | |
| 6 | rs3020391 | 152276923 | G | |  | 0.89 | | **0.04** |  | | 0.89 | | 0.07 |  | | 0.89 | | **0.03** |  |  | |
| 6 | rs3003921 | 152279514 | A | |  | 0.89 | | **0.03** |  | | 0.92 | | 0.16 |  | | 0.91 | | **0.04** |  |  | |
| 6 | rs3020401 | 152283044 | G | |  | 0.95 | | 0.25 |  | | 0.92 | | 0.11 |  | | 0.94 | | 0.15 |  |  | |
| 6 | rs985191 | 152283458 | C | |  | 1.03 | | 0.63 |  | | 0.94 | | 0.40 |  | | 1.00 | | 0.96 |  |  | |
| 6 | rs3003925 | 152284458 | G | |  | 0.89 | | **0.03** |  | | 0.87 | | **0.02** |  | | 0.88 | | **0.007** |  |  | |
| 6 | rs2982688 | 152285122 | G | |  | 0.90 | | **0.02** |  | | 0.92 | | 0.10 |  | | 0.91 | | **0.02** |  |  | |
| 6 | rs985695 | 152286705 | A | |  | 0.96 | | 0.47 |  | | 0.92 | | 0.16 |  | | 0.95 | | 0.28 |  |  | |
| 6 | rs2347869 | 152287295 | C | |  | 0.90 | | **0.02** |  | | 0.92 | | 0.09 |  | | 0.91 | | **0.02** |  |  | |
| 6 | rs2347871 | 152292660 | G | |  | 0.90 | | **0.01** |  | | 0.92 | | 0.10 |  | | 0.91 | | **0.02** |  |  | |
| 6 | rs2347872 | 152292672 | A | |  | 0.86 | | **0.01** |  | | 0.92 | | 0.24 |  | | 0.89 | | **0.02** |  |  | |
| 6 | rs3020325 | 152293905 | G | |  | 0.91 | | **0.02** |  | | 0.92 | | 0.09 |  | | 0.91 | | **0.02** |  |  | |
| 6 | rs2982683 | 152298435 | A | |  | 1.05 | | 0.28 |  | | 1.07 | | 0.18 |  | | 1.06 | | 0.18 |  |  | |
| 6 | rs726281 | 152302578 | G | |  | 0.91 | | 0.05 |  | | 0.95 | | 0.31 |  | | 0.93 | | 0.09 |  |  | |
| 6 | rs728524 | 152303437 | G | |  | 1.03 | | 0.84 |  | | 1.20 | | 0.28 |  | | 1.11 | | 0.46 |  |  | |
| 6 | rs9397463 | 152304328 | A | |  | 1.01 | | 0.92 |  | | 0.94 | | 0.36 |  | | 0.98 | | 0.72 |  |  | |
| 6 | rs926777 | 152305047 | A | |  | 0.90 | | **0.03** |  | | 0.91 | | 0.10 |  | | 0.91 | | **0.03** |  |  | |
| 6 | rs2982684 | 152306204 | A | |  | 0.77 | | **0.0005** |  | | 0.89 | | 0.15 |  | | 0.82 | | **0.003** |  | **intron 4** | |
| 6 | rs9371236 | 152306346 | G | |  | 1.03 | | 0.84 |  | | 1.20 | | 0.28 |  | | 1.11 | | 0.46 |  |  | |
| 6 | rs3020407 | 152307261 | G | |  | 0.91 | | 0.05 |  | | 0.96 | | 0.40 |  | | 0.93 | | 0.08 |  |  | |
| 6 | rs2144025 | 152307706 | A | |  | 0.90 | | 0.09 |  | | 0.99 | | 0.87 |  | | 0.94 | | 0.23 |  |  | |
| 6 | rs7743290 | 152309132 | C | |  | 0.92 | | 0.10 |  | | 0.97 | | 0.55 |  | | 0.93 | | 0.14 |  |  | |
| 6 | rs9340944 | 152313718 | A | |  | 1.00 | | 0.98 |  | | 1.02 | | 0.77 |  | | 1.00 | | 0.99 |  |  | |
| 6 | rs722208 | 152322885 | G | |  | 0.92 | | 0.09 |  | | 0.99 | | 0.92 |  | | 0.95 | | 0.19 |  |  | |
| 6 | rs13216134 | 152328484 | G | |  | 0.82 | | **0.004** |  | | 0.90 | | 0.18 |  | | 0.85 | | **0.009** |  |  | |
| 6 | rs1569788 | 152328616 | G | |  | 0.92 | | 0.08 |  | | 1.00 | | 0.94 |  | | 0.94 | | 0.18 |  |  | |
| 6 | rs9340958 | 152330673 | A | |  | 1.08 | | 0.29 |  | | 1.01 | | 0.92 |  | | 1.04 | | 0.53 |  |  | |
| 6 | rs9340969 | 152332130 | G | |  | 0.92 | | 0.10 |  | | 0.99 | | 0.92 |  | | 0.95 | | 0.20 |  |  | |
| 6 | rs13203975 | 152333104 | A | |  | 0.82 | | **0.004** |  | | 0.92 | | 0.26 |  | | 0.86 | | **0.01** |  |  | |
| 6 | rs9340978 | 152333945 | A | |  | 1.12 | | 0.19 |  | | 1.07 | | 0.47 |  | | 1.09 | | 0.25 |  |  | |
| 6 | rs3020418 | 152345162 | A | |  | 0.94 | | 0.22 |  | | 0.96 | | 0.42 |  | | 0.95 | | 0.21 |  |  | |
| 6 | rs9478265 | 152348901 | A | |  | 1.04 | | 0.64 |  | | 1.18 | | 0.11 |  | | 1.09 | | 0.28 |  |  | |
| 6 | rs6941835 | 152356270 | G | |  | 0.81 | | **0.01** |  | | 0.93 | | 0.42 |  | | 0.86 | | **0.03** |  |  | |
| 6 | rs2982712 | 152358179 | G | |  | 0.96 | | 0.30 |  | | 0.97 | | 0.60 |  | | 0.96 | | 0.35 |  |  | |
| 6 | rs3020434 | 152358940 | A | |  | 1.03 | | 0.54 |  | | 1.02 | | 0.78 |  | | 1.03 | | 0.62 |  |  | |
| 6 | rs3020365 | 152367993 | A | |  | 0.97 | | 0.47 |  | | 1.01 | | 0.90 |  | | 0.98 | | 0.64 |  |  | |
| 6 | rs3020366 | 152368758 | G | |  | 0.96 | | 0.34 |  | | 1.00 | | 0.98 |  | | 0.97 | | 0.50 |  |  | |
| 6 | rs3020368 | 152371190 | A | |  | 1.08 | | 0.28 |  | | 1.00 | | 1.00 |  | | 1.04 | | 0.57 |  |  | |
| 6 | rs9383962 | 152375362 | A | |  | 0.82 | | **0.005** |  | | 0.87 | | 0.07 |  | | 0.84 | | **0.006** |  | **intron 5** | |
| 6 | rs6932864 | 152376475 | A | |  | 0.83 | | **0.006** |  | | 0.87 | | 0.08 |  | | 0.85 | | **0.006** |  |  | |
| 6 | rs6913408 | 152378112 | A | |  | 0.91 | | 0.44 |  | | 0.72 | | **0.04** |  | | 0.84 | | 0.13 |  |  | |
| 6 | rs2273206 | 152382311 | A | |  | 0.83 | | **0.006** |  | | 0.87 | | 0.08 |  | | 0.85 | | **0.006** |  |  | |
| 6 | rs2273207 | 152382325 | G | |  | 0.81 | | **0.007** |  | | 0.93 | | 0.43 |  | | 0.86 | | **0.03** |  |  | |
| 6 | rs2207396 | 152382382 | A | |  | 1.05 | | 0.31 |  | | 1.08 | | 0.18 |  | | 1.06 | | 0.20 |  |  | |
| 6 | rs3778082 | 152387664 | A | |  | 0.84 | | **0.008** |  | | 0.87 | | 0.08 |  | | 0.85 | | **0.008** |  |  | |
| 6 | rs3020375 | 152389968 | C | |  | 0.96 | | 0.33 |  | | 0.99 | | 0.88 |  | | 0.97 | | 0.46 |  |  | |
| 6 | rs12199102 | 152392561 | A | |  | 0.98 | | 0.81 |  | | 1.04 | | 0.70 |  | | 1.00 | | 0.98 |  |  | |
| 6 | rs9479190 | 152393112 | G | |  | 0.84 | | **0.01** |  | | 0.88 | | 0.09 |  | | 0.86 | | **0.01** |  |  | |
| 6 | rs3822990 | 152405965 | A | |  | 0.81 | | **0.009** |  | | 0.93 | | 0.38 |  | | 0.86 | | **0.03** |  |  | |
| 6 | rs3020382 | 152412137 | G | |  | 1.07 | | 0.24 |  | | 1.04 | | 0.54 |  | | 1.05 | | 0.30 |  |  | |
| 6 | rs2982900 | 152414992 | A | |  | 1.27 | | **0.006** |  | | 1.11 | | 0.29 |  | | 1.20 | | **0.02** |  |  | |
| 6 | rs9341052 | 152416625 | G | |  | 0.99 | | 0.90 |  | | 1.07 | | 0.50 |  | | 1.03 | | 0.72 |  |  | |
| 6 | rs3778099 | 152418575 | G | |  | 0.85 | | **0.02** |  | | 0.88 | | 0.13 |  | | 0.87 | | **0.02** |  |  | |
| 6 | rs2228480 | 152420095 | A | |  | 0.99 | | 0.87 |  | | 1.06 | | 0.34 |  | | 1.02 | | 0.76 |  |  | |
| 6 | rs3798577 | 152421130 | G | |  | 1.02 | | 0.70 |  | | 1.02 | | 0.66 |  | | 1.02 | | 0.69 |  |  | |
| 6 | rs3798758 | 152421854 | A | |  | 0.75 | | **0.02** |  | | 0.78 | | 0.08 |  | | 0.77 | | **0.02** |  |  | |
| 6 | rs2747648 | 152422335 | G | |  | 0.99 | | 0.91 |  | | 0.96 | | 0.75 |  | | 0.97 | | 0.79 |  |  | |
| 6 | rs9341077 | 152423128 | G | |  | 1.00 | | 0.98 |  | | 1.08 | | 0.54 |  | | 1.03 | | 0.78 |  |  | |
| 6 | rs2813544 | 152425582 | G | |  | 1.05 | | 0.39 |  | | 0.97 | | 0.64 |  | | 1.02 | | 0.69 |  |  | |
| 6 | rs910416 | 152432902 | G | |  | 1.04 | | 0.33 |  | | 1.08 | | 0.10 |  | | 1.06 | | 0.15 |  |  | |

^1^ Chromosome, ^2^ Base pair position, ^3^ Minor allele, ^4^ Odds ratio, ^5^ P-value.
