# Supplementary material for: Polymorphisms in Genes of Relevance for Oestrogen and Oxytocin Pathways and Risk of Barrett’s Oesophagus and Oesophageal Adenocarcinoma: A Pooled Analysis from the BEACON Consortium
Source: PLoS One. 2015 Sep 25;10(9):e0138738. doi: 10.1371/journal.pone.0138738 (PMC4583498; doi:10.1371/journal.pone.0138738)
Supplement: S5 Table — (DOCX) [file pone.0138738.s006.docx]

**Supporting Information**

**S5 Table.** Single nucleotide polymorphisms (SNPs) results for the oxytocin receptor gene (*OXTR*) and risk of Barrett’s oesophagus in the total sample. P-values in bold are significant.

|  | | | | | **Barrett’s oesophagus** | |  | |
| --- | --- | --- | --- | --- | --- | --- | --- | --- |
| **CHR^1^** | **SNP** | **BP^2^** | **A1^3^** |  | **OR^4^** | **P^5^** |  | **Position** |
| 3 | rs237875 | 8782406 | A |  | 0.98 | 0.64 |  |  |
| 3 | rs237877 | 8782887 | A |  | 0.99 | 0.77 |  |  |
| 3 | rs6777088 | 8786487 | G |  | 1.02 | 0.64 |  |  |
| 3 | rs13087941 | 8787220 | G |  | 1.07 | 0.16 |  |  |
| 3 | rs13093809 | 8788096 | G |  | 1.12 | 0.06 |  |  |
| 3 | rs11476 | 8788198 | A |  | 1.01 | 0.83 |  |  |
| 3 | rs7629329 | 8788336 | G |  | 1.01 | 0.82 |  |  |
| 3 | rs2324728 | 8792728 | A |  | 1.06 | 0.24 |  |  |
| 3 | rs237884 | 8793585 | G |  | 1.06 | 0.21 |  |  |
| 3 | rs6770632 | 8793724 | A |  | 1.05 | 0.29 |  |  |
| 3 | rs1042778 | 8794545 | A |  | 1.05 | 0.26 |  |  |
| 3 | rs237885 | 8795543 | A |  | 0.91 | **0.02** |  |  |
| 3 | rs11706648 | 8796547 | C |  | 1.05 | 0.33 |  |  |
| 3 | rs237887 | 8797042 | G |  | 0.90 | **0.02** |  |  |
| 3 | rs2268490 | 8797085 | A |  | 0.93 | 0.24 |  |  |
| 3 | rs237888 | 8797095 | G |  | 1.15 | 0.11 |  |  |
| 3 | rs918316 | 8798181 | G |  | 1.10 | 0.26 |  |  |
| 3 | rs4686301 | 8798586 | A |  | 1.05 | 0.34 |  |  |
| 3 | rs2268491 | 8800398 | A |  | 0.90 | 0.12 |  |  |
| 3 | rs2254298 | 8802228 | A |  | 0.90 | 0.13 |  |  |
| 3 | rs237889 | 8802483 | A |  | 0.97 | 0.47 |  |  |
| 3 | rs11131149 | 8802851 | A |  | 1.06 | 0.18 |  |  |
| 3 | rs237895 | 8807423 | A |  | 0.91 | **0.02** |  |  |
| 3 | rs2268495 | 8807535 | A |  | 0.97 | 0.53 |  |  |
| 3 | rs237897 | 8808285 | A |  | 0.90 | **0.01** |  |  |
| 3 | rs237899 | 8808515 | A |  | 1.14 | **0.003** |  |  |
| 3 | rs237902 | 8809184 | A |  | 1.13 | **0.008** |  |  |
| 3 | kgp3933398 | 8809222 | A |  | 1.07 | 0.31 |  |  |
| 3 | rs237911 | 8810008 | G |  | 1.01 | 0.82 |  |  |
| 3 | rs2301261 | 8810896 | A |  | 0.93 | 0.35 |  |  |
| 3 | rs6777726 | 8813494 | A |  | 0.91 | 0.30 |  |  |
| 3 | rs180789 | 8813927 | G |  | 1.14 | **0.005** |  |  |
| 3 | rs6443206 | 8820075 | G |  | 0.95 | 0.27 |  |  |
| 3 | rs75775 | 8820732 | A |  | 0.96 | 0.51 |  |  |
| 3 | rs9860869 | 8820740 | C |  | 0.99 | 0.88 |  |  |

^1^ Chromosome, ^2^ Base pair position, ^3^ Minor allele, ^4^ Odds ratio, ^5^ P-value
