# Supplementary material for: Polymorphisms in Genes of Relevance for Oestrogen and Oxytocin Pathways and Risk of Barrett’s Oesophagus and Oesophageal Adenocarcinoma: A Pooled Analysis from the BEACON Consortium
Source: PLoS One. 2015 Sep 25;10(9):e0138738. doi: 10.1371/journal.pone.0138738 (PMC4583498; doi:10.1371/journal.pone.0138738)
Supplement: S6 Table — (DOCX) [file pone.0138738.s007.docx]

**Supporting Information**

**S7 Table.** Single nucleotide polymorphisms (SNPs) results for the oxytocin receptor gene (*OXTR*) and risk of Barrett’s oesophagus ***in males.*** P-values in bold are significant.

|  | | | | | **Barrett’s oesophagus** | |  | | |
| --- | --- | --- | --- | --- | --- | --- | --- | --- | --- |
| **CHR^1^** | **SNP** | **BP^2^** | **A1^3^** |  | **OR^4^** | **P^5^** | |  | **Position** |
| 3 | rs237875 | 8782406 | A |  | 1.02 | 0.72 | |  |  |
| 3 | rs237877 | 8782887 | A |  | 0.96 | 0.43 | |  |  |
| 3 | rs6777088 | 8786487 | G |  | 1.02 | 0.69 | |  |  |
| 3 | rs13087941 | 8787220 | G |  | 1.05 | 0.35 | |  |  |
| 3 | rs13093809 | 8788096 | G |  | 1.11 | 0.12 | |  |  |
| 3 | rs11476 | 8788198 | A |  | 1.00 | 0.92 | |  |  |
| 3 | rs7629329 | 8788336 | G |  | 1.01 | 0.91 | |  |  |
| 3 | rs2324728 | 8792728 | A |  | 1.05 | 0.38 | |  |  |
| 3 | rs237884 | 8793585 | G |  | 1.06 | 0.32 | |  |  |
| 3 | rs6770632 | 8793724 | A |  | 1.04 | 0.47 | |  |  |
| 3 | rs1042778 | 8794545 | A |  | 1.09 | 0.09 | |  |  |
| 3 | rs237885 | 8795543 | A |  | 0.87 | **0.004** | |  |  |
| 3 | rs11706648 | 8796547 | C |  | 1.06 | 0.28 | |  |  |
| 3 | rs237887 | 8797042 | G |  | 0.89 | **0.02** | |  |  |
| 3 | rs2268490 | 8797085 | A |  | 0.88 | 0.07 | |  |  |
| 3 | rs237888 | 8797095 | G |  | 1.09 | 0.41 | |  |  |
| 3 | rs918316 | 8798181 | G |  | 1.14 | 0.15 | |  |  |
| 3 | rs4686301 | 8798586 | A |  | 1.07 | 0.21 | |  |  |
| 3 | rs2268491 | 8800398 | A |  | 0.87 | 0.06 | |  |  |
| 3 | rs2254298 | 8802228 | A |  | 0.86 | 0.05 | |  |  |
| 3 | rs237889 | 8802483 | A |  | 0.96 | 0.43 | |  |  |
| 3 | rs11131149 | 8802851 | A |  | 1.08 | 0.11 | |  |  |
| 3 | rs237895 | 8807423 | A |  | 0.88 | **0.008** | |  |  |
| 3 | rs2268495 | 8807535 | A |  | 0.99 | 0.87 | |  |  |
| 3 | rs237897 | 8808285 | A |  | 0.87 | **0.004** | |  |  |
| 3 | rs237899 | 8808515 | A |  | 1.16 | **0.003** | |  |  |
| 3 | rs237902 | 8809184 | A |  | 1.18 | **0.001** | |  |  |
| 3 | kgp3933398 | 8809222 | A |  | 1.10 | 0.19 | |  |  |
| 3 | rs237911 | 8810008 | G |  | 1.01 | 0.94 | |  |  |
| 3 | rs2301261 | 8810896 | A |  | 0.90 | 0.25 | |  |  |
| 3 | rs6777726 | 8813494 | A |  | 0.92 | 0.38 | |  |  |
| 3 | rs180789 | 8813927 | G |  | 1.13 | **0.03** | |  |  |
| 3 | rs6443206 | 8820075 | G |  | 0.94 | 0.28 | |  |  |
| 3 | rs75775 | 8820732 | A |  | 0.94 | 0.40 | |  |  |
| 3 | rs9860869 | 8820740 | C |  | 1.02 | 0.81 | |  |  |

^1^ Chromosome, ^2^ Base pair position, ^3^ Minor allele, ^4^ Odds ratio, ^5^ P-value.
